# Construction and comparison of different vehicles for heterologous gene expression in *Zymomonas mobilis*

**DOI:** 10.1111/1751-7915.14381

**Published:** 2024-01-24

**Authors:** Gerrich Behrendt, Maria Vlachonikolou, Helga Tietgens, Katja Bettenbrock

**Affiliations:** ^1^ Analysis and Redesign of Biological Networks, Max Planck Institute for Dynamics of Complex Technical Systems Magdeburg Germany

## Abstract

*Zymomonas mobilis* has the potential to be an optimal chassis for the production of bulk chemicals derived from pyruvate. However, a lack of available standardized and characterized genetic tools hinders both efficient engineering of *Z. mobilis* and progress in basic research on this organism. In this study, a series of different shuttle vectors were constructed based on the replication mechanisms of the native *Z. mobilis* plasmids pZMO1, pZMOB04, pZMOB05, pZMOB06, pZMO7 and p29191_2 and on the broad host range replication origin of pBBR1. These plasmids as well as genomic integration sites were characterized for efficiency of heterologous gene expression, stability without selection and compatibility. We were able to show that a wide range of expression levels could be achieved by using different plasmid replicons. The expression levels of the constructs were consistent with the relative copy numbers, as determined by quantitative PCR. In addition, most plasmids are compatible and could be combined. To avoid plasmid loss, antibiotic selection is required for all plasmids except the pZMO7‐based plasmid, which is stable also without selection pressure. Stable expression of reporter genes without the need for selection was also achieved by genomic integration. All modules were adapted to the modular cloning toolbox Zymo‐Parts, allowing easy reuse and combination of elements. This work provides an overview of heterologous gene expression in *Z. mobilis* and adds a rich set of standardized genetic elements to an efficient cloning system, laying the foundation for future engineering and research in this area.

## Graphical abstract

In this study, a series of shuttle vectors compatible with modular cloning were constructed based on the replication mechanisms of the native *Z. mobilis* plasmids pZMO1, pZMOB04, pZMOB05, pZMOB06, pZMO7 and p29191_2 and on the broad host range replication origin of pBBR1. These plasmids as well as genomic integration sites were characterized for efficiency of heterologous gene expression, stability without selection and compatibility.

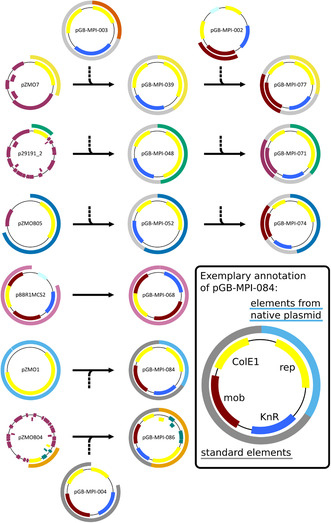

## INTRODUCTION

*Zymomonas mobilis* is an ethanologenic alphaproteobacterium with very compelling attributes as a chassis organism for industrial use. *Z. mobilis* is facultative anaerobic and unique in using the Entner–Doudoroff pathway preferably anaerobically (Swings & De Ley, 1977), resulting in a titer, relative productivity, and yield with respect to ethanol far surpassing those of other ethanologenic microbes (Rogers et al., 1982). However, limitations in the usability and efficiency of genetic tools are still holding back the utilization of *Z. mobilis* in industrial applications (Kalnenieks et al., 2020).The wild type strains are limited in their substrate and product range (Swings & De Ley, 1977) and in their tolerance to inhibitory substances (Franden et al., 2009), characteristics that should be altered through changes on the genetic level to achieve economically viable processes by metabolic engineering.

Shuttle vectors, being able to replicate in a cloning host and the host of interest, are very useful tools in microbiology (Nora et al., 2019; Struhl et al., 1979). Several studies have focused on the creation of new shuttle vectors for *Z. mobilis* (Arvanitis et al., 2000; Browne et al., 1984; Cao et al., 2016; So et al., 2014), mostly based on genetic elements from a variety of native plasmids present in the different *Z. mobilis* strains. However, a missing standard of design and the limited public availability of the constructs obstruct the easy and at the same time the sophisticated choice of the right shuttle vector for a given research task (Kalnenieks et al., 2020). Alternatively, plasmids based on broad host range vectors like pBBR1MCS2 (Kovach et al., 1995) or pRSF1010 (Scherzinger et al., 1984) are often used for engineering of *Z. mobilis*. However, these constructs are rather large or seem to achieve only relatively low levels of gene expression. In addition, in some studies heterologous expression units were integrated into the chromosome (or a native plasmid) (Hu et al., 2023; Li et al., 2022). However, the specific sites of integration into the genome were not carefully investigated with respect to the potential they hold for such endeavours.

The Zymo‐Parts toolbox aims at providing tested and reusable genetic parts for genetic engineering of *Z. mobilis* (Behrendt et al., 2022). This also includes the provision of different plasmids that vary with respect to copy number and can be used e.g. for heterologous gene expression in *Z. mobilis* and that allow for the introduction and stable maintenance of two or even more plasmids in one cell. To identify promising candidates, the literature on genetic tools for heterologous gene expression in *Z. mobilis* was closely examined and is summarized here. Based on this literature study, a selection of plasmids and acceptor sites for homologous recombination was adapted to the Golden Gate modular cloning system Zymo‐Parts (Behrendt et al., 2022) to ease future research on *Z. mobilis* by providing standardized and thoroughly analysed genetic elements. The Zymo‐Parts system is based on the modularization of functional genetic elements (Weber et al., 2011) and the efficient assembly of these elements by Cut‐Ligation using type IIS restriction enzymes like *Bsa*I and *Bbs*I (Engler et al., 2009). This allows multiple genetic elements to be assembled in a specified order into a backbone, containing the replication elements. Six additional replication systems based on native *Z. mobilis* plasmids and one from a broad host range plasmid were added to the Zymo‐Parts toolbox and were combined with different antibiotic resistance cassettes. In addition, homology arms for recombination into defined genomic loci were added.

The plasmids and genomic loci were characterized concerning expression strength of heterologous genes as well as stability. Additionally, plasmid compatibility and relative copy number was determined. This work also gives an overview on available plasmids for strain design in *Z. mobilis* and is intended to provide a basis for future engineering projects, either by using the Zymo‐Parts plasmids and modules presented in this work or by creating new plasmids, based on the provided sequences and data.

## EXPERIMENTAL PROCEDURES

### Strains and media

All plasmid construction was performed using *Escherichia coli* NEB5α from NewEnglandBiolabs (NEB), grown in LB_0_ medium (10 g/L tryptone, 5 g/L yeast extract, 5 g/L NaCl) at 37°C with shaking. Experiments for the characterization of shuttle vectors were performed with *Z. mobilis* strain ATCC 31821 (ZM4). Cultivations were performed at 30°C in complex medium (ZM: Bacto peptone 10 g/L, yeast extract 10 g/L, glucose 20 g/L). Kanamycin and chloramphenicol were used at 100 μg/mL, spectinomycin was used at 150 μg/mL. Bacto agar 15 g/L was added for solid media.

### Construction of plasmids

Plasmids were constructed according to the design of the Zymo‐Parts Toolbox (Behrendt et al., 2022). The different DNA fragments were amplified by PCR using Q5 DNA Polymerase (NEB) according to the manufacturer's recommendations. The corresponding primers and assembly procedures are listed in File S1. Sequences of all plasmids described in this publication are available as annotated Genbank files in an Edmond repository under https://doi.org/10.17617/3.J7QUR3.

Six new plasmids were constructed based on previous published work and sequence analysis (see Section 3). The corresponding sequences containing the putative plasmid replicons were amplified by PCR using genomic DNA of strains ATCC 10988, NCIMB 11163 or ATCC 29191. Primers used for amplification are listed in File S1. The PCR products were ligated to the other modules (pUC‐origin, antibiotic resistance cassette, etc.) via Cut‐Ligation as described previously.

### Introduction of plasmids into *Z. mobilis*

Electroporation was performed as described in Behrendt et al. (2022). For conjugation, plasmids were first transformed into the *E. coli* donor strain ST18 (Thoma & Schobert, 2009). Transformants were then incubated in LB_0_ with δ‐aminolevulinic acid (50 mg/L) and the respective antibiotic overnight with shaking at 37°C, while *Z. mobilis* acceptor strains were incubated in ZM (with antibiotic if necessary) with 2% (w/v) glucose overnight at 30°C without shaking. Subsequently, fresh cultures were inoculated from the overnight cultures, and grown to mid‐exponential growth phase under the same conditions. 1 mL of culture from donor and acceptor strain with an OD_600_ of approximately 0.5 was mixed and centrifuged at 17,000 × *g* for 30 s. The supernatant was discarded and the pellet was resuspended in the remaining liquid and then dropped onto a ZM plate containing δ‐aminolevulinic acid (50 mg/L) and the respective antibiotics, if applicable, and incubated overnight at 30°C. Bacteria were detached from the plate with an inoculation loop and suspended in 1 mL of ZM, pelleted at 17,000 × *g*, and resuspended in 1 mL of ZM. Finally, the bacteria were incubated for 2 h at 30°C and 100 μL of different dilutions of the culture was plated out onto ZM plates with antibiotics.

### Flow cytometry analysis

Quantification of mCherry and eGFP fluorescence by flow cytometry was performed in a CyFlow Space flow cytometer (Sysmex) as described in Behrendt et al. (2022). The number of biological replicates is indicated in the respective figure legends. Data analysis was done with Flowing Software 2 (Turku Bioscience) and CytoExploreR (Hammill, 2021).

### Determination of plasmid copy number by qPCR

Plasmid copy number was determined by qPCR by relative quantification compared to the chromosome. The PCR was performed with Takyon No Rox SYBR MasterMix dTTP Blue (Eurogentec) in 20 μL reaction volume. Cultures were diluted to an OD_600_ of 0.1, 0.05, 0.01 and 0.005, respectively, and 0.8 μL of the respective dilution was used directly as template in the PCR reaction. Primers were used at a concentration of 2 ng/μl. Primer pairs for the amplification of chromosomal DNA were directed to a region near *oriC* (Fuchino, Wasser, & Soppa, 2021) (oriqF3 + oriqR3) and to ZMO0028 (ZMO0028qF + ZMO0028qR). For amplification of plasmid DNA, primers were directed to the kanamycin (KanrqF + KanrqR) or chloramphenicol resistance genes (CamrqF + CamrqR), respectively (For primer sequences see File S1). Real Time PCR reactions were performed in a Rotor‐Gene 6000 (Qiagen). Amplification conditions were: 95°C for 10 min, followed by 40 cycles at 95°C for 15 s and 60°C for 1 min. A negative control without template for each primer pair was included in each PCR run. The amplification efficiency was comparable for all primer pairs. Quantification was performed by relative quantification of the plasmid DNA versus chromosomal DNA using the Δ*C*t method without efficiency correction.

### Determination of plasmid stability

To determine the stability of the different constructs without selective pressure, each construct was first introduced into *Z. mobilis* ATCC 31821. For each construct, six positive colonies were used to each inoculate 5 mL of ZM media with 2% (w/v) glucose and kanamycin, and the cultures were incubated in tubes at 30°C for 24 h without agitation. An aliquot of each culture was analysed for fluorescence by flow cytometry and the cultures were diluted 1000‐fold in fresh medium without antibiotics. This process was repeated for 5 days. In this setup, each culture produces approximately 10 generations in a 24 h cycle, resulting in approximately 50 generations overall without selection. The starting culture grown with antibiotic selection was designated “day 0” and the following cultures without antibiotic were designated “day 1” to “day 5”. As a reference, ATCC 31821 carrying a plasmid without reporter gene was used to gate non‐fluorescent cells (Gate A, including 99% of reference cells) and fluorescent cells (Gate B). The ratio of counts (counts of Gate B)/(counts of Gate A + counts of Gate B) was used to express the proportion of fluorescent cells (for the reference the ratio was 0.01).

### Analysis of plasmid compatibility

To analyse the compatibility of the different shuttle vectors in *Z. mobilis* strain ATCC 31821, carrying either pZP599, pZP1131, pZP595, pZP605, pZP1133 or pZP465 was conjugated with ST18 with either pZP1190, pZP1149, pZP1186, pZP1146, pZP1152 or pZP1188, so that each of the replication systems was combined with each other.

### Genomic integration

All plasmids used for chromosomal integration are described in detail in File S1. 400–800 bp long regions flanking the genomic locus of interest were amplified from genomic DNA of ATCC 31821 and inserted into lvl 1 acceptors of the Zymo‐Parts toolbox (Behrendt et al., 2022). To construct suicide plasmids used for genomic integration, these homology arms were combined with a kanamycin resistance cassette and an *mcherry* expression unit (Pstrong100k*‐rbs10k‐*mcherry*‐TsoxR), as previously described for locus ZMO0028 (Behrendt et al., 2022). The resulting plasmids pZP1206, pZP1210, pZP1214, pZP1218 and pZP1222 were electroporated into ATCC 31821. Selection for integration was done on plates with kanamycin.

## RESULTS AND DISCUSSION

### Shuttle vectors for *Z. mobilis*

The design of plasmids for heterologous gene expression in *Z. mobilis* as well as other niche organisms is generally not a coordinated effort (Silva‐Rocha et al., 2013). An overview of systems used for heterologous gene expression in *Z. mobilis* can be found in Table 1 (with a focus on shuttle vectors), Table 2 (with a focus on broad host range vectors) and Table 3 (with focus on genomic insertion sites).

**TABLE 1:** Comprehensive overview of shuttle vectors used in published work on *Z. mobilis*.

Origin of the plasmid	Purpose	Plasmid line	Host strain	Overexpressed genes	Publication
ATCC 10988 pZM2 (assumed to be pZMOB06)	New shuttle vector	pZA22	NRRL B‐14023		Misawa et al. (1986), *Agricultural and Biological Chemistry*
	Production of β‐Carotene	pZA22	NRRL B‐14023	*crtB*, *crtE*,*crtI* and *crtY* of *Erwinia uredovora*	Misawa et al. (1991), *Applied and Environmental Microbiology*
ATCC 10988 pZMOB06 (assumed)	Xylose assimilation, pentose phosphate pathway	pZB5	NRRL B‐14023	*xylA*/*xylB*, *tal*/*tktA*	Zhang et al. (1995), *Science*
	Xylose assimilation, pentose phosphate pathway	pZM27	ATCC 31821	*xylA*/*xylB*, *tal*/*tktA*	Agrawal et al. (2011), *Biotechnology and Bioengineering*
	Embden–Meyerhof–Parnas pathway	pZM27	ATCC 31821	*pfk*, *fbp*, *tpi*	Chen et al. (2013), *Applied Biochemistry and Biotechnology*
ATCC 10988 pZM2 (assumed to be pZMOB06)	Improved thermotolerance	pZA22	TISTR 548	Thermotolerance candidates	Anggarini et al. (2020), *Frontiers in Microbiology*
	Stress‐tolerant ethanol fermentation	pZA22	TISTR 548	*groESL*	Kaewchana et al. (2021), *Applied Microbiology and Biotechnology*
ATCC 10988 pZMOB06	2,3‐butanediol production	pEZ15Asp	ATCC 31821 8b	Different 2,3‐BDO biosynthesis pathway genes	Yang et al. (2016), *Biotechnology for Biofuels*
	Promoter characterization	pEZ15Asp	ATCC 31821	*mcherry* and *egfp*	Yang et al. (2019), *Biotechnology for Biofuels*
	CRISPR‐cas characterization	pEZ15Asp	ATCC 31821	crDNA	Shen et al. (2019), *Microbial Cell Factories*
ATCC 10988 pZMOB06	CRISPR‐cas characterization	pEZ15Asp	ATCC 31821	crDNA	Zheng et al. (2019), *Nucleic Acids Research*
	Genome editing characterization	pEZ15Asp	ATCC 31821 (ZM4mrr)	crDNA	Sui et al. (2021), *Biotechnology for Biofuels*
	Genome editing characterization	pEZ15Asp	ATCC 31821 (ZM4mrr)	crDNA	Wang et al. (2021), *Journal of Genetics and Genomics*
	Isobutanol production	pEZ15Asp	ATCC 31821	Different isobutanol biosynthesis pathway genes	Qiu et al. (2020), *Biotechnology for Biofuels*
	Ethylene production	pEZ15Asp	ATCC 31821	*ethylene‐forming enzyme*	He et al. (2021), *Applied Microbiology and Biotechnology*
	Researching self‐flocculation	pEZ15Asp	ATCC 31821	ZMO1083/2–1085 of ZM401	Cao et al. (2022), *Applied and Environmental Microbiology*
	Ethanol tolerance	pEZ15Asp	ATCC 31821	*hfq*	Tang et al. (2022), *Frontiers in Bioengineering and Biotechnology*
ATCC 10988 pZMOB06, ATCC 31821 pZM39	Poly‐3‐hydroxybutyrate production	pEZ15Asp, pEZ39p	ATCC 31821	*phaCAB* and several other genes	Li et al. (2022), *Green Chemistry*
ATCC 10988 pZMOB06	Acetoin production	pEZ15Asp	ATCC 31821	*aldC*, *als*, *noxE*	Bao et al. (2023), *Fermentation*
	Lactate production, Isobutanol production	pYL6	ATCC 31821	*ldhA*, *alsS*, *ilvC*, *ilvD*, *kivd*, *adhA*	Liu et al. (2020), *Metabolic Engineering*
	Characterization of regulatory elements	pZP	ATCC 31821	*mcherry*, *egfp* and *ldhA*	Behrendt et al. (2022), *ACS Synthetic Biology*
	Lactate production	pEZ15Asp	ATCC 31821	*ldhA of Leuconostoc mesenteroides*	Hu et al. (2023), *Frontiers in Bioengineering and Biotechnology*
NCIMB 11163 pZMO7	New shuttle vector, protein–protein binding interactions.	pZ7C	NCIMB 11163, ATCC 29191	*acpP*, *kdsA*, *holC*, and *hfq*	So et al. (2014), *BMC Microbiology*
NRRL B‐14023* *oriC*, pZZM401, pZZM402	New shuttle vector	pSUZM1, pSUZM2, pSUZM3	NRRL B‐14023*, ATCC 29191, CICC 10232 and CICC 10225		Cao et al. (2016), *Electronic Journal of Biotechnology*
NRRL B‐14023 pZZM401	Recombineering system	pSUZM2	ATCC 31821	*recE*, *recE588* and *recT*	Wu et al. (2017), *Electronic Journal of Biotechnology*
	Genome engineering	pSUZM2	ATCC 31821	*cas9*	Cao et al. (2017), *Bioscience, Biotechnology, and Biochemistry*
ATCC 29191 pNSW1 (ZM6100)	New shuttle vector	pNSW601	ATCC 29191		Browne et al. (1984), *Plasmid*
	New shuttle vector	pNSW301	ZM6100		Strzelecki et al. (1987), *Plasmid*
	Stability determination, new derivatives	pNSW301	ZM6100		Strzelecki et al. (1990), *Plasmid*
ATCC 29191 pNSW2 (15.5 kb) (ZM6100)	New shuttle vector	pOK2	ZM6100		Cho et al. (1989), *Applied Microbiology and Biotechnology*
ATCC 10988 pZMO1, ATCC 10988 pZMO2	New shuttle vector	pDA11, pDA21	ATCC 10988, NRRL B‐14023		Arvanitis et al. (2000), *Plasmid*
ATCC 10988 pZMO2	New shuttle vector	pDS212	CUl		Afendra and Drainas (1987), *Journal of General Microbiology*
ATCC 10988 pZM3 (assumed to be pZMOB05)	New shuttle vector	pZA31‐33	NRRL B‐14023		Tonomura et al. (1986), *Agricultural and Biological Chemistry*

**TABLE 2:** Overview of broad host range vectors used in *Z. mobilis*.

Origin of the plasmid	Purpose	Plasmid line	Host strain	Overexpressed genes	Publication
*oriV* from RSF1010	Optimized vector for *Z. mobilis*	pLOI193	NRRL B‐14023	*beta‐galactosidase*	Conway et al. (1987), *Applied and Environmental Microbiology*
	Overexpression of *gfo*, *ppc*, *bglA*, *mdh*, and *fdh1*	pHW20a	ATCC 31821, ATCC 29191	*gfo*, *ppc*, *bglA*, *mdh*, and *fdh1*	Dong et al. (2011), *Biotechnology and Bioengineering*
	Resistance against formate	pHW20a	ATCC 31821	*formate dehydrogenase*	Dong et al. (2013), *Biotechnology and Bioengineering*
	Resistance against aldehyde inhibitors	pHW20a	ATCC 31821	ZMO1721	Yi et al. (2021), *Applied Biochemistry and Biotechnology*
	Resistance against phenolic aldehydes	pHW20a	ATCC 31821	ZMO1162	Yi et al. (2022), *Bioprocess and Biosystems Engineering*
	Researching self‐flocculation	pHW20a	ATCC 31822	ZMO1055 of ATCC 31821 and ZM401, *bcsA* of ZM401	Cao et al. (2022), *Applied and Environmental Microbiology*
	Researching biofilm formation	pHW20a	ATCC 31822	*pfs* and *luxS* of *E. coli*	Cao et al. (2023), *Frontiers in Bioengineering and Biotechnology*
*oriV* of pBBR1MSC1	Production of Xylonic Acid	pB1	ATCC 31821	Several xylose dehydrogenase genes	Herrera et al. (2021), *Microorganisms*
	Production of fructooligosaccharides	pB1	ATCC 31821	β‐fructofuranosidase from *Schwanniomyces occidentalis*, *sacB*	Braga et al. (2022), *Applied Microbiology and Biotechnology*
	Regulated redirection of central carbon flux	pZmpdc	ATCC 31821	Inducible expression of the *pyruvate decarboxylase*	Liu et al. (2020), *Metabolic Engineering*
	Isoprene production	pBBR1	ATCC 31821	*dxs2*, *ispG*, *ispH*, *ispS*, *IDI*	Khana et al. (2023), *mSystems*

**TABLE 3:** Overview of chromosomal and native plasmid integration used in *Z. mobilis*.

Locus	Method	Purpose	Host strain	Overexpressed genes	Publication
ZMOp41x016 and between ZMO1268/ZMO2012	Transposon	Xylose assimilation, pentose phosphate pathway	ATCC 31821	*xylA/xylB*, *tal/tktA*	Zhang et al. (2007), U.S. Patent No. 7223575
ZMO0256, ZMO1083‐85	Homologous recombination	Proof of concept	ATCC 31281	*crtIBE*	Lal et al. (2019), *Frontiers in Microbiology*
ZMO0038	Native CRISPR‐Cas	Poly‐3‐hydroxybutyrate production	ATCC 31821	*phaCAB*	Li et al. (2022), *Green Chemistry*
ZMO0150	Homologous recombination	Malic acid production	ATCC 31821	*Ec mae*	Khandelwal et al. (2022), *Journal of Biotechnology*
ZMO0056 (downstream)	Tn7 transposon	CRISPR interference	ATCC 31821	*Spy dcas9*	Banta et al. (2020), *Applied and Environmental Microbiology*
ZMO0038, ZMO1360, ZMO1650	Native CRISPR‐Cas	Lactate production		native *pdc* and *ldhA of L. mesenteroides*	Hu et al. (2023), *Frontiers in Bioengineering and Biotechnology*

Literature suggests that so far the replication mechanisms of 11 native *Z. mobilis* plasmids were utilized for the construction of shuttle vectors. However, 22 of the 32 publications listed in Table 1 rely on pZMOB06 as origin for replication in *Z. mobilis*, making it the most prominent basis for shuttle vectors in *Z. mobilis* research.

For some of the shuttle vectors described, it is difficult to determine which native plasmid forms the basis. This is especially true for older constructs that rely solely on restriction maps for the identification of plasmids. In addition, some plasmids have been renamed after their initial sequencing. For example, pZM3 from ATCC 10988, the basis of the shuttle vectors pZA31‐33 (Tonomura et al., 1986), is most likely pZMOB05, based on size and restriction map. Some publications refer to a 2.7 kb plasmid of ATCC 10988 as the source of their vector (Agrawal et al., 2011; Zhang et al., 1995) or pZM2 (Misawa et al., 1986, 1991), which is most likely pZMOB06. Another difficulty is the confusion between the strains NRRL B‐14023 and ATCC 31821, as the plasmids pZZM401 to pZZM405 (GenBank: CP001881.1–CP001885.1) were thought to be the native plasmids of ATCC 31821, but are actually derived from NRRL B‐14023 (Yang et al., 2018). This highlights the importance of checking carefully which strain and plasmid one is actually working with, preferably using sequencing (Coton et al., 2006) and the need to determine the plasmid sequences of older constructs.

In some cases, the described shuttle vectors appear promising but have only been used by the groups that created them, an example being pSUZM2 (Cao et al., 2016, 2017; Wu et al., 2017). Most studies use only a single shuttle vector for heterologous gene expression, although there are some examples of shuttle vectors being used in combination with broad host range vectors (Liu et al., 2020), genome insertions (Li et al., 2022) or in combination with other shuttle vectors (Li et al., 2022).

### Construction of new shuttle vectors

Until now, all shuttle vectors of the genetic toolbox Zymo‐Parts were based on the replicon of pZMOB06 (Genebank: NC_017185; ATCC 10988) for replication in *Z. mobilis*. In the study at hand, six additional plasmid replication systems that are based on the replication systems of the native *Z. mobilis* plasmids pZMO1 (Genebank: NC_011363; ATCC 10988), pZMOB04 (Genebank: NC_017184; ATCC 10988), pZMOB05 (Genebank: NC_017182; ATCC 10988), pZMO7 (Genebank: NC_019300; NCIMB 11163), and p29191_2 (Genebank: CP003706; ATCC 29191) were added to Zymo‐Parts. In addition, the replication system of the broad host range plasmid pBBR1MCS2 (Kovach et al., 1995) was used.

**FIGURE 1:**
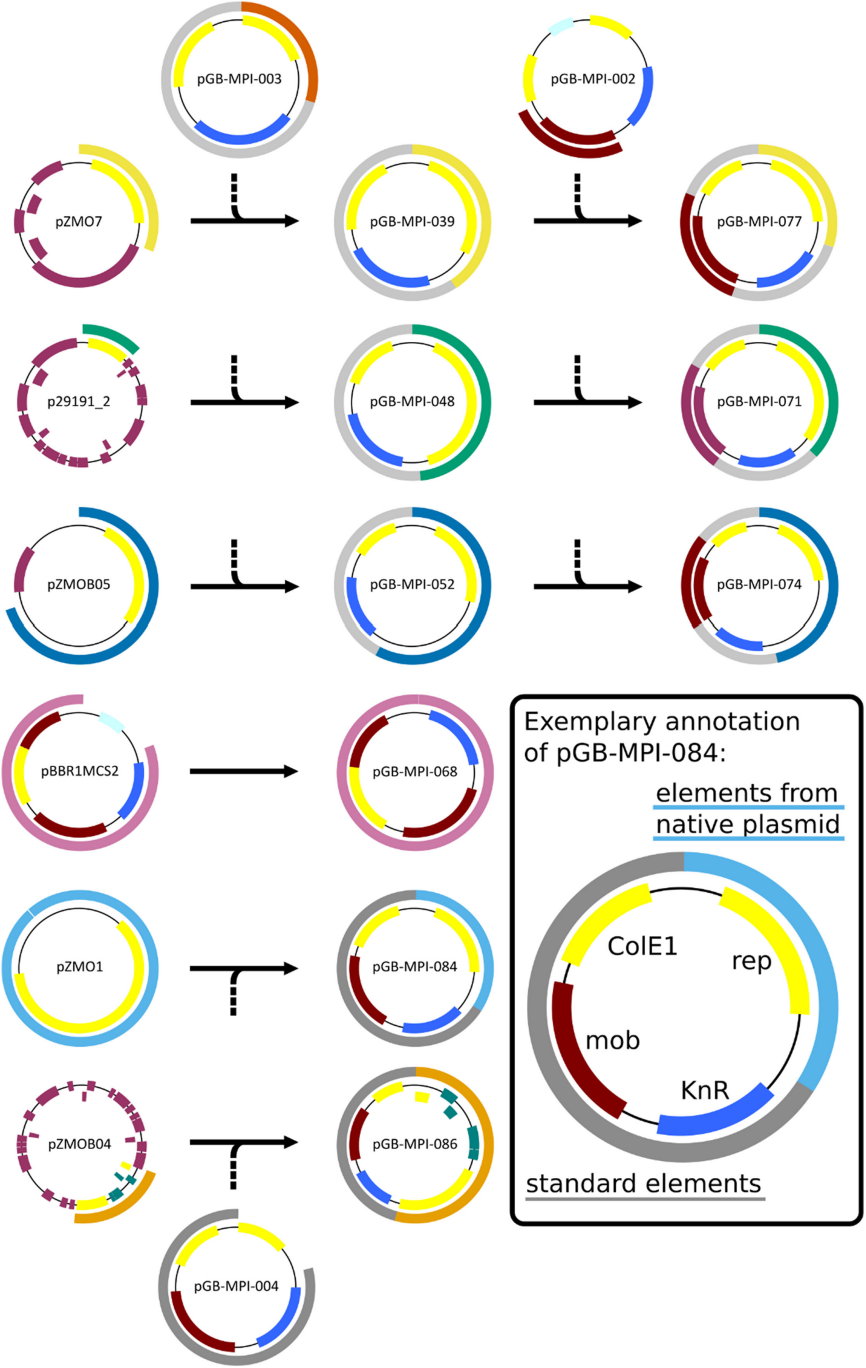
A simplified graphical overview of the plasmid lineage created from native *Z. mobilis* plasmids and existing Zymo‐Parts vectors. Plasmids pGB‐MPI‐039/048/052 were created from pGB‐MPI‐003 (kanamycin resistance and ori ColE1) and equipped with a mobilization element from pGB‐MPI‐002 to create plasmids pGB‐MPI‐077/071/074, while pGB‐MPI‐084/086 were created directly with a mobilization element from pGB‐MPI‐004, which is derived from pBBR1MCS2. pGB‐MPI‐068 was derived from pBBR1MCS2 but elements were rearranged to match the other toolbox elements. In the figure, the outer rings correspond to the regions amplified from the native plasmids, with grey shading for parts of the backbone required for selection and replication in *E. coli* and specific colour coding for the native plasmids and their derivatives. On the inner rings, open reading frames and the ColE1 ori (yellow) are annotated by blocks; in addition, putative replication‐associated elements are shown in yellow, kanamycin resistance in blue, and other elements in dark red. The mobilization elements as well as the regions flanking them are also coloured dark red. The size of the elements is chosen relative to the respective size of the plasmid.

Sequences from native plasmids were selected based on previous work (Arvanitis et al., 2000; Browne et al., 1984; Liu et al., 2020; Misawa et al., 1986; So et al., 2014; Strzelecki et al., 1987; Tonomura et al., 1986; Yang et al., 2016). All plasmids based on sequences from native *Z. mobilis* plasmids are additionally equipped with the ColE1 origin of replication for cloning and maintenance in *E. coli*, and with an antibiotic resistance cassette for selection. As transformation efficiencies by electroporation varied widely, a mobilization element from pBBR1MCS2 (Kovach et al., 1995) was added and used for conjugation‐based transfer. All cloning was based on Golden Gate cloning or *Sma*I Cut‐Ligation. A graphical overview of the shuttle vector design is shown in Figure 1.

The broad host range vector pBBR1MCS2 (Kovach et al., 1995) is frequently utilized in *Z. mobilis* (Braga et al., 2022; Herrera et al., 2021; Liu et al., 2020). pGB‐MPI‐068 carries the *rep*, *oriV* and *mob* elements of pBBR1MCS2 and was further modified to create acceptors fitting the Zymo‐Parts system.

Strain ATCC 10988 carries eight native plasmids, many of which have been previously studied or used to construct shuttle vectors. In this study we used four of these plasmids: pZMO1, pZMOB04, pZMOB05 and pZMOB06. In literature, pZMOB06‐derived shuttle vectors are by far the most frequently used plasmids for heterologous gene expression in *Z. mobilis* (see Table 1). pZMOB06 was used for shuttle vector construction at least three times independently, first for pZA22 (Misawa et al., 1986), later for pEZ15Asp (Yang et al., 2016), the most often cited shuttle vector line for *Z. mobilis*, and then for pYL6 (Liu et al., 2020). With its size of 2.7 kb, pZMOB06 is rather small, and an approximately 900 bp region carrying the *rep* gene (ZMOB_RS09665) and probably also *oriV* was used to create pEZ15Asp, pYL6 and pGB‐MPI‐003 from the Zymo‐Parts toolbox (Behrendt et al., 2022). The plasmids pZMO1 and pZMOB05 show some similarity as a larger part of their sequence is filled with non‐coding palindromic regions. pZMO1 and the closely related (Chen et al., 2018) pZMO1A of NCIMB 11163 have both been used to construct shuttle vectors (Arvanitis et al., 2000; So et al., 2014). pZMOB05, previously referred to as pZM3, forms the basis of the shuttle vectors pZA31, pZA32, and pZA33 (Tonomura et al., 1986). In this work, elements of pZMO1 and pZMOB05 were combined as parts of pGB‐MPI‐084, and pGB‐MPI‐052 and its mobilizable derivative pGB‐MPI‐074, respectively. To our knowledge, pZMOB04 has never before been used as the base of a shuttle vector. A 3864 bp region ranging from Zmob_1850 to Zmob_1855 (Pappas et al., 2011) was used to construct pGB‐MPI‐086. This region contains a gene encoding a helix‐turn‐helix domain‐containing protein (Marchler‐bauer et al., 2016) (pfam13730) protein (Zmob_1855). Such proteins are often associated to replication. The region also contains two putative toxin‐antitoxin systems (Zmob_1852/1853 and Zmob_1854/1855).

The shuttle vector pZ7C is based on pZMO7 from NCIMB 11163 (So et al., 2014). Reportedly pZ7C is stable for 50 generations without selection (So et al., 2014), but there are no reports about its use for engineering purposes after its initial publication. pGB‐MPI‐039 and pGB‐MPI‐077 (with *mob*) are based on a 1.4 kb region of pZMO7, which includes the putative rep HS589_RS00010 and its flanking regions and may therefore be similar to pZ7C (Genebank: NC_019300.1).

A 2 kb region of p29191_2 of ATCC 29191 was used to construct pGB‐MPI‐048 and pGB‐MPI‐071 (with *mob*). Early *Z. mobilis* shuttle vectors pNSW301 and pNSW601 were based on pNSW1 of ATCC 29191 substrain ZM6100, which is most likely identical to p29191_2. The region chosen from p29191_2 was motivated by homologies to the region taken from pZZM401 of NRRL B‐14023 for the creation of pSUZM2 (Cao et al., 2016).

Table 4 summarizes the plasmids used in this work.

**TABLE 4:** Overview of plasmids used in this work.

Acronym	Ori	Resistance	Reporter	Mob
pGB‐MPI‐039	pZMO7	kanamycin	−	−
pGB‐MPI‐077	pZMO7	kanamycin	−	+
pZP605	pZMO7	kanamycin	*mcherry*	−
pZP1152	pZMO7	chloramphenicol	*egfp*	+
pGB‐MPI‐048	p29191_2	kanamycin	−	−
pGB‐MPI‐071	p29191_2	kanamycin	−	+
pZP595	p29191_2	kanamycin	*mcherry*	−
pZP1146	p29191_2	chloramphenicol	*egfp*	+
pGB‐MPI‐052	pZMOB05	kanamycin	−	−
pGB‐MPI‐074	pZMOB05	kanamycin	−	+
pZP599	pZMOB05	kanamycin	*mcherry*	−
pZP1149	pZMOB05	chloramphenicol	*egfp*	+
pZP1133	pZMOB04	kanamycin	*mcherry*	+
pGB‐MPI‐086	pZMOB04	kanamycin		+
pZP1188	pZMOB04	chloramphenicol	*egfp*	+
pGB‐MPI‐068	pBBR1MCS2	kanamycin	−	+
pZP1135	pBBR1MCS2	kanamycin	*mcherry*	+
pZP1190	pBBR1MCS2	chloramphenicol	*egfp*	+
pZP1131	pZMO1	kanamycin	*mcherry*	+
pGB‐MPI‐084	pZMO1	kanamycin	−	+
pZP1186	pZMO1	chloramphenicol	*egfp*	+
pZP465	pZMOB06	kanamycin	*mcherry*	−
pZP1191	pZMOB06	chloramphenicol	*egfp*	+

### Comparison of shuttle vector mediated expression strength

**FIGURE 2:**
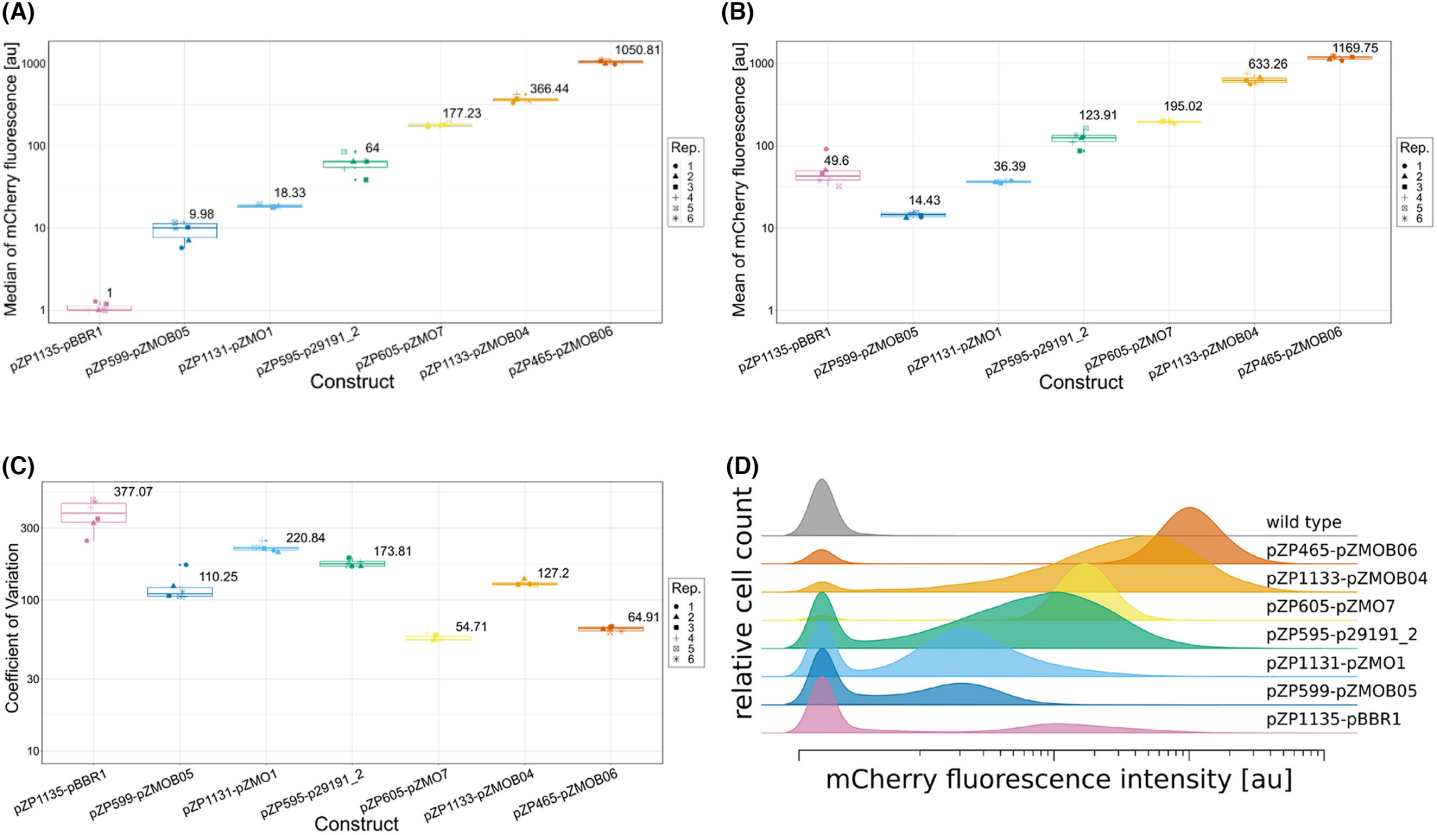
mCherry expression in cultures carrying different shuttle vectors. Box plots showing (A) the medians, (B) the means and (C) the distribution (as CV) of mCherry fluorescence intensity using the same expression cassette in different shuttle vectors. Each point represents the mCherry fluorescence from a single cultivation of ATCC 31821 with the respective plasmids pZP465 (pZMOB06 ori), pZP595 (p29191_2 ori), pZP599 (pZMOB05 ori), pZP605 (pZMO7 ori), pZP1131 (pZMO1 ori), pZP1133 (pZMOB04 ori) and pZP1135 (pBBR1MCS2 ori). A total of six biological replicates per construct were measured. The median of all measurements per construct is displayed next to each box. (D) Example histograms of the distribution of mCherry fluorescence intensity for 50,000 cells from ATCC 31821 with the respective expression plasmid.

In addition to the expression plasmid pZP465, already included in the Zymo‐Parts toolbox (Behrendt et al., 2022), six new expression plasmids derived from the plasmids described above (Figure 1), were analysed based on mCherry expression. All plasmids share the same regulatory elements, namely the promoter Pstrong100k*, the ribosomal binding site rbs10k, and the terminator TsoxR. The plasmids were introduced into ATCC 31821 and analysed for reporter gene expression. For each construct at least 50,000 cells of an overnight culture were analysed with respect to fluorescence intensity (Figure 2A,B) and fluorescence distribution (Figure 2C,D).

The data shown in Figure 2 demonstrate that a wide range of expression levels can be achieved by using different origins of replication. The range between the average fluorescence intensity of the constructs is about 100‐fold and the selection of a suitable expression plasmid can thus be an alternative to the manipulation of gene expression levels by using different promotors (Behrendt et al., 2022) or rbs (Yang et al., 2019). The high‐expressing plasmids tended to have a unimodal distribution of expression levels, while the low‐expressing plasmids seemed to follow a bimodal expression pattern. Bimodal distributions were also observed for pRSF1010‐based replication systems in *E. coli* (Jahn et al., 2016). Since all plasmids share the same expression cassette it is tempting to speculate that the expression level is influenced by the copy number of the plasmid. In this case, low expression levels would imply low copy numbers averaged over the whole population of cells. Bimodal population distributions may therefore be related to the control of plasmid replication and segregation. However, also the efficiency of the selection by the antibiotic will impact this behaviour. For some replicons it may not be possible to ensure that all daughter cells receive a plasmid copy, especially not for low copy number plasmids. Notably, for plasmids derived from pBBR1, pZMO1 and p29191_2, a few transformants (<5%) showed a much higher fluorescence intensity and a much smaller variance within the population (data not shown) than the majority. It was not possible to isolate and retransform plasmids from these highly expressing colonies. This may indicate a tendency for chromosomal integration of these plasmids and should be taken into account when working with them. The mechanism and location of this potential integration are unknown.

Based on both the median and mean fluorescence detected, the derivative of pZMOB06 clearly showed the strongest mCherry expression. As this replicon is the most commonly used system in *Z. mobilis* research (see Table 1), this suggests that most projects aimed for strong overexpression. pZP1133 (pZMOB04 ori) achieved the second highest level of mCherry expression. Its backbone may therefore be a good alternative, allowing a slightly lower but still high level of expression.

As the antibiotic resistance mechanism might have strong influence on plasmid stability and the homogeneity of the cultures, variants carrying an *egfp* expression unit and either a resistance against kanamycin, chloramphenicol or spectinomycin were created and analysed in the same fashion as the original mcherry‐kanamycin variants (see Figures S1A–D, S2A–D and S3A–D in File S2 respectively). The relative fluorescence levels of the original high‐fluorescence creating replication systems based of pZMO7, pZMOB04 and pZMOB06 remained mostly unchanged, while the distribution of fluorescence intensity was strongly altered for variants based of pZMO1, pZMOB05, p29191_2 and pBBR1.

### Determination of plasmid copy number by qPCR

**FIGURE 3:**
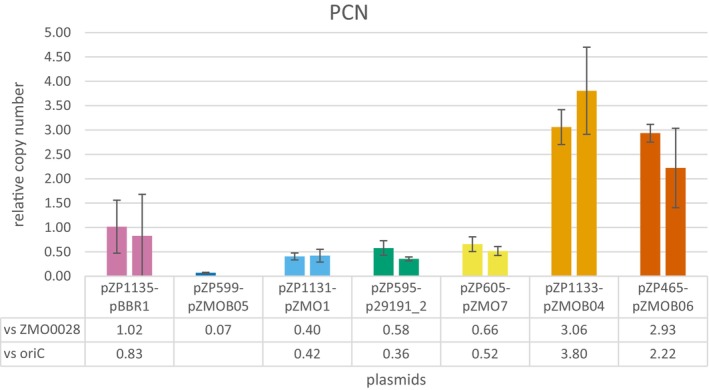
Relative plasmid copy numbers. Shown is the relative amount of the plasmid marker (kanamycin resistance) with respect to the chromosomal locus ZMO0028 (left bar) and *oriC* (right bar) as determined by the Δ*C*_T_ method.

Plasmid copy number (PCN) is an important factor in selecting the best vector for a given application. As absolute quantification (copies per cell) is difficult, PCN was determined in relation to the chromosome (Figure 3) by comparing the amount of a plasmid marker (the kanamycin resistance) to two chromosomal markers (ZMO0028 and *oriC*) using quantitative PCR. Remarkably, five plasmids had a relative PCN of 1 or even less than 1 and only two plasmids had copy numbers higher than the chromosome. This was unexpected as in *E. coli* and most other hosts the number of plasmid copies per cell is at least equal to or significantly higher than the number of chromosomes (Jahn et al., 2016). This could be explained by recent reports about the polyploidy of *Z. mobilis* (Brenac et al., 2019; Fuchino, Chan, et al., 2021; Fuchino, Wasser, & Soppa, 2021).

With a relative PCN per cell of 0.07 for pZP599 (pZMOB05 ori) to 3.8 for pZP1133 (pZMOB04 ori) and an assumed 12 to 16 chromosome copies per cell for ATCC 31821 (Fuchino, Wasser, & Soppa, 2021), PCN for the tested constructs would vary between 1 and 60. This range is similar to PCNs for common expression plasmids in *E. coli*, which range from 2 to 40 copies per cell (Jahn et al., 2016). We used replicons of native plasmids from *Z. mobilis*. To be stably maintained in *Z. mobilis* these plasmids need to place a low metabolic burden on the cell which might be one reason for the low copy numbers observed (Brockhurst & Harrison, 2022).

The determined copy numbers (Figure 3) are consistent with the determined mCherry expression levels (Figure 2). This is reasonable as all constructs share the same elements of mCherry expression. Notably, low PCN is associated with high variation in target gene expression within a population, which is consistent with existing hypotheses on plasmid distribution (Jahn et al., 2015; Summers, 1991). However, little variation was observed for the pZMOB05‐based derivative pZP599 despite its low copy number. This may indicate a tight control of its copy number and partitioning similar to P1 and F plasmid replication and partitioning in *E. coli* (Gordon et al., 1997). It should be noted that the fluorescence distributions shown in Figure 2D indicate that for plasmids based on pBBR1, pZMOB05, pZMO1 and p29191_2 a there is a population without plasmid. In this case the low relative PCN determined represents an average of the whole cultuure and hence from both populations and does not reflect the PCN in a specific cell.

### Determination of plasmid stability

**FIGURE 4:**
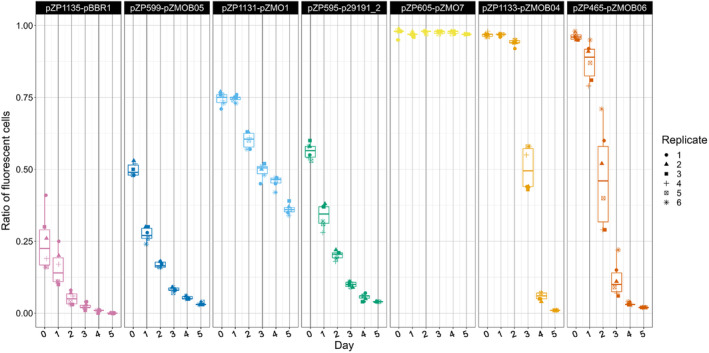
Box plot showing time course data of the percentage of fluorescent cells of ATCC 31821 carrying different shuttle vectors. Day 0 shows data from the first cultivation containing kanamycin for plasmid selection, from day 1 onwards no kanamycin was added.

An important property for the application of a plasmid is its stability. The stability of the seven different vectors was therefore tested in ATCC 31821 (Figure 4) by culturing them for approximately 50 generations in the absence of antibiotic selection. At each inoculation of a new culture samples were analysed by flow cytometry, and the proportion of red fluorescent cells in the total population was measured.

In the absence of antibiotic selection pressure, the proportion of fluorescent cells decreased steadily for all constructs except for pZP605 (pZMO7 ori). The drop‐off of the proportion of fluorescent cells is different for the different plasmids. While pZM1131, for example, declines rather slowly, pZM1133 appears to decline more rapidly. For some constructs the reduction in the proportion of plasmid‐bearing cells is delayed and sets in after 1 or 2 days. For pZP605 even after 50 generations without antibiotic selection, almost all cells in the population expressed mCherry. This confirms the observation by So et al. (2014) who reported that their pZMO7‐based shuttle vector was stable for at least 50 generations. pZP605, unlike the derivative pZ7C (So et al., 2014), does not carry any of the genes of pZMO7 other than the putative replication‐associated gene. The stability appears to be associated with the replication gene and may be ensured by host cell factors. In bulk chemical production, fermentation costs are an important factor in profitability (Straathof, 2011) and the ability to dispense antibiotics for production may be a factor favouring the use of pZMO7‐derived backbones over other plasmids in industrial applications of *Z. mobilis*. However, plasmid stability will be influenced by the metabolic burden the construct imposes on the cells and needs to be checked for the specific application.

pZP1133 (pZMOB04 ori) was the second most stable vector without selection, based on the percentage of fluorescent cells after 2 days without antibiotic selection, followed by pZP1131 (pZMO1 ori) and then pZP465 (pZMOB06 ori). Cultures containing the low copy number vectors pZP595 (p29191_2 ori), pZP599 (pZMOB05ori), pZP1131 (pZMO1 ori), and pZP1135 (pBBR1MCS2 ori) showed a low proportion of non‐fluorescent cells even in the presence of the antibiotic (day 0). This is partly due to the low level of fluorescence they generate, which overlaps with the background fluorescence of cells not expressing mCherry but could also be related to inefficient plasmid partitioning that produces cells without plasmid. How significant this effect is depends on the efficiency of antibiotic selection. Another factor is the high variance of the fluorescence intensity within the population for these four plasmids (see Figure 2C,D). The proportion of mCherry expressing cells decreased rapidly for these constructs, with the exception of the pZP1131 (pZMO1 ori), which decreased more slowly and still showed around 37.5% fluorescent cells after 5 days without antibiotic selection. The reason for this higher plasmid stability is unknown but will most probably be inherent to the replication and partitioning mechanisms.

**FIGURE 5:**
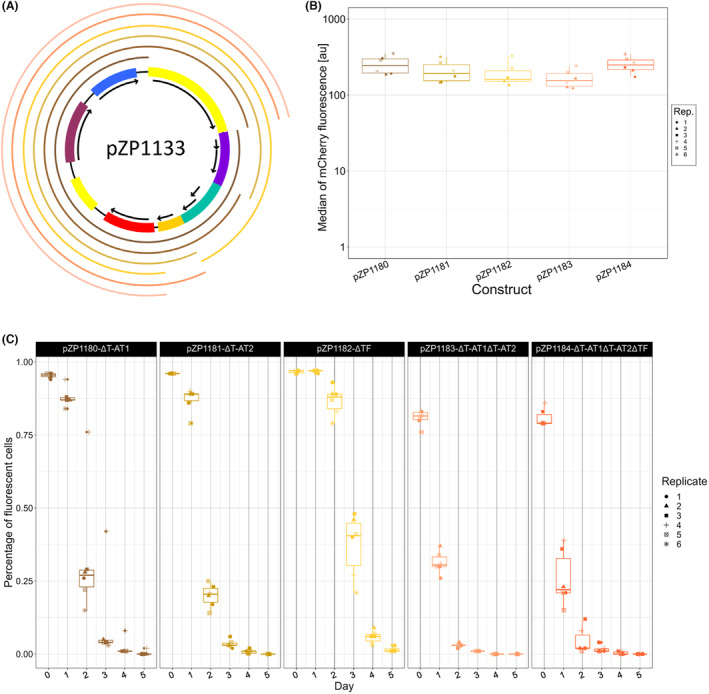
Stability of the deletion variants of pZP1133. (A) Schematic sketch of pZP1133. Functional regions are colour coded: Yellow – replication, Purple – T‐AT1, Green – T‐AT2, Orange – TF, Red – mcherry TU, Dark Red – mobility element, Blue – kanamycin resistance. The incomplete outer circles represent the deletion variants pZP1179 to pZP1184 (from inside to outside). Since pZP1179 could not be introduced into ATCC 31821 it is not shown in (B and C). (B) Median fluorescence of the derivatives of pZP1133 measured by flow cytometry. (C) Box plots showing the development of the proportion of fluorescent cells in the population over time for cultivations without antibiotic selection. Day 0 shows data from the first culture containing kanamycin for plasmid selection, from day 1 onwards no kanamycin was added.

pZP1133, based on pZMOB04, represents a completely new shuttle vector line with no prior knowledge. Computational annotation suggests that it harbours two toxin‐antitoxin systems. The suspected T‐AT are encoded by ZMOB_RS10035 and ZMOB_RS09570, as well as ZMOB_RS09565 and ZMOB_RS09560 (Kouvelis et al., 2011) (characterized as HicA family toxin, HicB family antitoxin, RelB/DinJ family antitoxin and RelE/ParE family toxin, in that order by automated annotation based on NC_017184). For simplicity, the T‐ATs will be referred to as T‐AT1 and T‐AT2, respectively. Six deletion variants were generated (Figure 5A) and tested for expression strength (Figure 5B) and stability (Figure 5C). We deleted the predicted replication associated protein (pZP1179), the first T‐AT (pZP1180), the second T‐AT (pZP1181), the transcriptional regulator (pZP1182), both T‐ATs (pZP1183) and both T‐ATs and the transcriptional regulator (pZP1184). The variant lacking the replication‐associated protein could not be introduced into *Z. mobilis*, confirming that this element (Zmob_1855) is responsible for replication. All deletion variants showed similar levels of fluorescence and fluorescence distributions in the presence of kanamycin.

Data in Figure 5C show that the two putative T‐AT systems of pZMOB04 do not completely stabilize the plasmid pZP1133 in ATCC 31821, but that both systems contribute to stability. Variants lacking both T‐AT systems (pZP1183 and pZP1184) already start with a signification proportion of cells without the plasmid and lose the plasmid rapidly, while variants with only one of the T‐AT systems start with a higher proportion of plasmid‐containing cells (pZP1180 and pZP1181) but again the proportion of plasmid containing cells decreases more quickly than for pZP1133 or its variant containing both T‐AT systems (pZP1182). In summary, the main effect of the T‐AT systems in this shuttle vector line is to increase the proportion of plasmid‐containing cells under selective conditions.

### Compatibility of shuttle vectors

For some biotechnological applications it may be advantageous to have two plasmids simultaneously in one strain. For example, when expressing multiple genes, it is often advantageous to split the genetic load between two plasmids. This allows a better and separate control of the expression of the different genes and reduces the size of the plasmid. To test the compatibility of the different plasmids introduced in this work, variants with a chloramphenicol resistance cassette and *egfp* were constructed. ATCC 31821 was first transformed with the plasmid variants carrying *mcherry* and kanamycin resistance and the resulting strains were then transformed with the *egfp* and chloramphenicol resistance variants. An overview of compatibility is given in Table 5. Most plasmids were compatible with each other. This observation was not surprising since most *Z. mobilis* strains carry multiple native plasmids which must be compatible with each other. Selected combinations of pZP465 (pZMOB06 ori) plus pZP1152 (pZMO7 ori, pZP465 (pZMOB06 ori) plus pZP1146 (p29191_2 ori), and pZP1146 (p29191_2 ori) plus pZP605 (pZMO7 ori) were tested for coexistence stability by culturing the respective strains for approximately 40 generations in the presence of both antibiotics. No change in the fluorescence intensity was observed (data not shown). For completeness, we also checked whether native plasmids were displaced by the shuttle vectors. However, we did not observe any changes in the native plasmid pool (data not shown).

**TABLE 5:** Compatibilities of the shuttle vectors in ATCC 31821.

	pBBR1MCS2 (pZP1135)	pZMOB05 (pZP599)	pZMO1 (pZP1131)	P29191_2 (pZP595)	pZMO7 (pZP605)	pZMOB04 (pZP1133)	pZMOB06 (pZP465)
pBBR1MCS2 (pZP1190)		√	√	√	√	N	√
pZMOB05 (pZP1149)			√	√	√	N	N
pZMO1 (pZP1186)				√	√	√	√
P29191_2 (pZP1146)					√	√	√
pZMO7 (pZP1152)						√	√
pZMOB04 (pZP1188)							√
pZMOB06 (pZP1191)							

*Note*: Columns show vectors coding for mcherry and kanamycin resistance, rows show vectors coding for egfp and chloramphenicol resistance. Compatibility is marked with “√”, if compatibility was not observed the combination is marked with “N”.

The strains carrying two plasmids were examined for the expression of both reporters by flow cytometry. No difference in the distribution of the mCherry fluorescence intensity was observed compared to the single plasmid analysis. However, for the plasmids pZP1190 (pBBR1 ori), pZP1149 (pZMOB05 ori) and pZP1146 (p29191_2 ori), the distribution of the eGFP fluorescence was not completely consistent with the relative expression levels of the corresponding mCherry derivatives. This could also be observed in strains carrying only one of the corresponding constructs. In particular, these plasmids showed increased levels of reporter gene expression (Figure S1 in File S2). The reasons for this are not clear but may be related to the use of different antibiotics for the selection or to differences in the DNA superfolding due to sequence differences. For example, a chloramphenicol‐dependent increase in the copy number of ColE1 and pMB1 plasmids is a well‐known phenomenon (Clewell, 1972). Since there is no detailed knowledge of the replication mechanisms of the different plasmids, this effect needs to be further elucidated.

It could hence be shown that most of the replication systems chosen for our study are compatible with each other and can be combined in *Z. mobilis*, if wanted. This makes the genetic engineering of *Z. mobilis* even more flexible. If wanted, two (or even three) constructs could be introduced instead of one large construct. This is advantageous because transformation efficiencies often decrease for large constructs and there is more freedom to adjust expression levels. It is also possible to combine stable with less stable plasmids which could be used in genetic engineering methods where specific genes like recombinase are needed only for a limited period of time.

### Integration into chromosomal loci and native plasmids

An alternative for heterologous gene expression from a vector is the insertion of expression units into chromosomal loci. This avoids problems with plasmid stability and the need for selection. We wanted to analyse the expression of heterologous genes when inserted into different chromosomal or native plasmid loci. For this, we chose four loci on the chromosome and one on the native plasmid pZM36 as integration sites motivated by previous work, namely the loci ZMO0038, ZMO0256, ZMO1083‐1085, ZMO1667 and ZMOp36x016. Locus ZMO0038 was previously used to insert and overexpress *phaCAB* for the production of poly‐3‐hydroxybutyrate (Li et al., 2022). It encodes a ribosomal protein, S30Ae/sigma 54 modulation protein, which was previously described as being non‐essential (Zheng et al., 2019). The lactate dehydrogenase of *Z. mobili*s, encoded by ZMO0256, has been also been described as dispensable (Strazdina et al., 2018) and was previously used to express *crtIEB* as a reporter system (Lal et al., 2019). The loci ZMO1083 to ZMO1085 encode *bcsABC*. The corresponding proteins are responsible for cellulose synthesis and are involved in the cellular aggregation of *Z. mobilis* (Jones‐Burrage et al., 2019). ZMO1167 encodes *ctpA*. The *ctpA* homologue ZZ6_1449 from ATCC 29191 may be involved in salt tolerance (Fuchino & Bruheim, 2020), making it an interesting candidate for genomic insertions. Finally, ZMOp36x016 is located on the native plasmid pZM36 and has no experimentally validated function. We chose this locus because in the xylose‐utilizing ATCC 31821 substrain 8b the *Peno_talB‐tktA‐cat* expression unit is inserted into this locus (Yang et al., 2018). Since 8b is known for its efficient utilization of xylose, we wanted to evaluate ZMOp36x016 as a target site in general.

**FIGURE 6:**
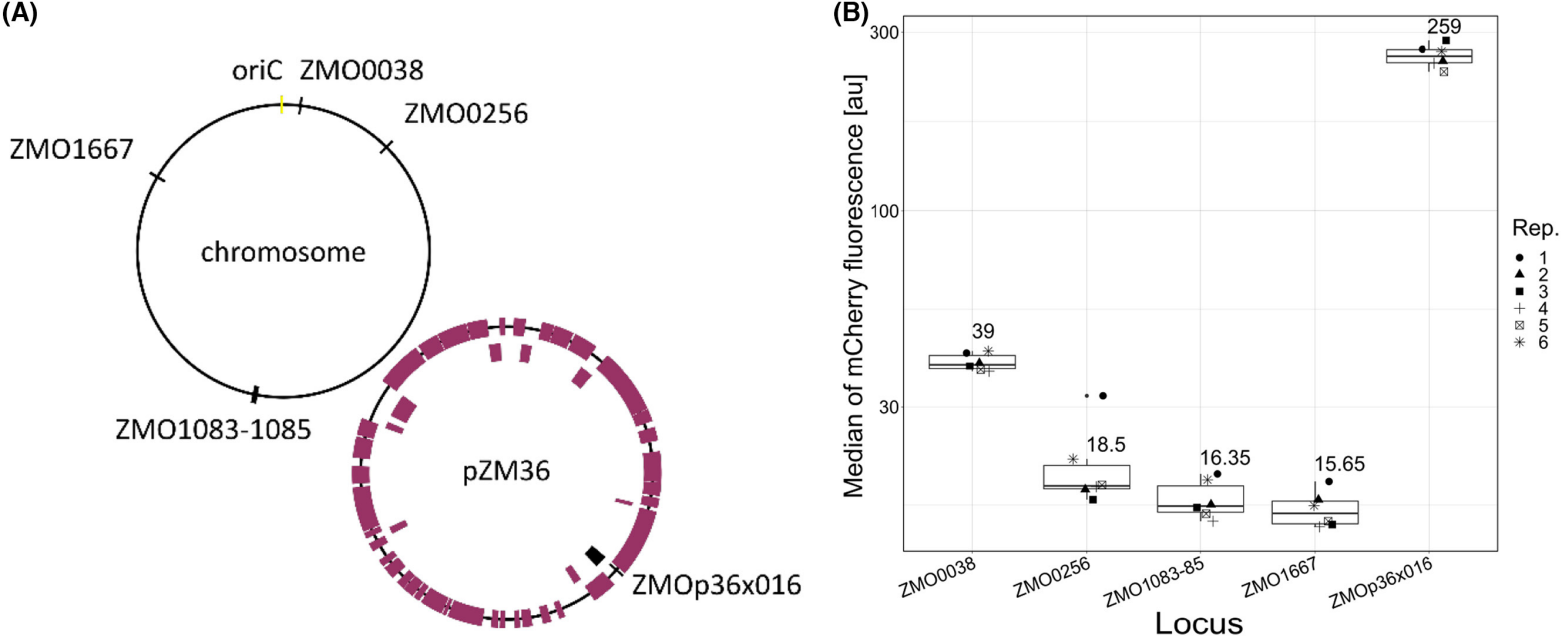
Integration of mCherry expression units into chromosomal loci and native plasmid. (A) Graphical overview of the position of the loci on the chromosome or the native plasmid pZM36. (B) Boxplot showing the median mCherry fluorescence determined for each insertion site by flowcytometry.

To determine the suitability of different loci on the chromosome and on the native plasmid pZM36 for heterologous gene expression, homology arms were selected to achieve homologous recombination at these sites. The respective upstream and downstream homology arms were combined with a kanamycin resistance gene and an *mcherry* expression unit (Pstrong100k*‐rbs10k‐*mcherry*‐TsoxR) to create suicide plasmids (ColE1 origin of replication) – all within the Zymo‐Parts toolbox – that provide the template for replacement of the respective locus with the kanamycin resistance cassette and the expression unit for *mcherry*. The positions of the insertion sites on the chromosome and on the native plasmid pZM36 are shown in Figure 6.

Integration of the mcherry expression unit into the chromosomal loci ZMO0256, ZMO1083‐85 and ZMO1667 resulted in similar levels of mCherry fluorescence, comparable to those obtained with the low copy expression plasmids. The locus ZMO0038 supported a slightly higher level of expression. This may be due to its proximity to the *oriC*, which has been reported to be present at a higher copy number (Brenac et al., 2019; Fuchino, Chan, et al., 2021; Fuchino, Wasser, & Soppa, 2021). Expression from ZMOp36x016 resulted in a much higher mCherry fluorescence intensity than from any of the chromosomal loci, although a low copy number of 1–2 per chromosome has been previously observed (Yang et al., 2018). An effect of cis regulatory effects on expression strength is unlikely as the expression directed by the combination of Pstrong100k* and rbs10k is the highest observed for regulatory elements (Behrendt et al., 2022). The reason for the high expression from this locus is unknown and needs further analysis.

**FIGURE 7:**
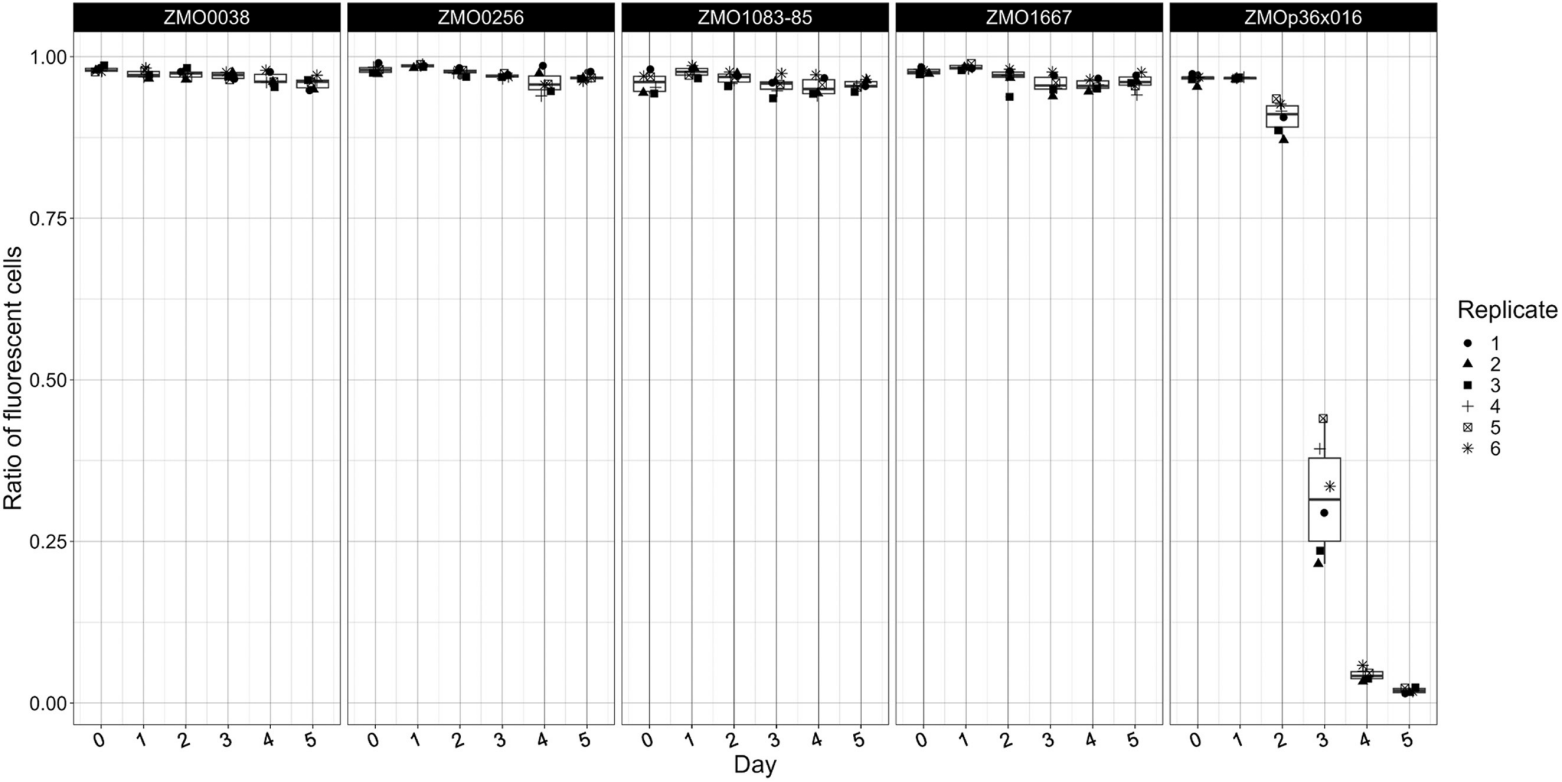
Stability of genomic integrations. The box‐plots show the percentage of fluorescent cells of ATCC 31821 with integration of *mcherry* expression units into different loci. Day 0 shows data from the starting culture containing kanamycin for selection integrands, from day 1 onwards no selection was applied.

For completeness, we performed stability assays for the chromosomal and episomal integrations (Figure 7). All tested chromosomal integrations were stable and almost 100% of the cells expressed *mcherry* after 5 days without antibiotic selection, while cultures with the integration in ZMOp36x016 became bimodal after 2 days. The fraction of non‐fluorescent cells increased from day three and after 5 days without selection hardly any expressing cells were left. This is similar to the stability of the shuttle vectors and again demonstrates that the shuttle vectors tested in this work behave similar to the corresponding native plasmids. It is most likely, that the insertion into the locus of pZM36 resulted in heterogeneous strains, carrying copies of pZM36 with the insertion cassette and copies of the wild type variant of the native plasmid in each cell. Without the selection for the edited variant the unaltered pZM36 obviously displaces the modified plasmid for unknown reason.

## CONCLUSION

This publication presents and analyses seven different shuttle vectors and chromosomal integration sites for their utility in heterologous gene expression in *Z. mobilis*. Six of the shuttle vectors are similar to previously characterized shuttle vectors, but have been reduced in size and made compatible with the Zymo‐Parts Toolbox or Golden Gate Cloning in general. The data collected for the constructs with respect to copy number, strength of heterologous gene expression, stability and compatibility will facilitate their usability and increase their value for *Z. mobilis* focused research. The analysed derivatives of pZMOB04, which has never been used for shuttle vector construction before, show characteristics suitable for heterologous gene expression and will be further explored as the basis for expression systems in our future research.

In summary, based on the data presented here, the available modularized backbones will allow researchers to select specifically one or more replication system(s) for their needs in basic research or metabolic engineering. Due to the ease of access and the high efficiency of Golden Gate cloning, it is possible to combine the vectors with all elements of the Zymo‐Parts Toolbox, allowing for systematic screening of different constructs. With the Zymo‐Parts toolbox a broad basis for the selection of suitable sets and combinations of genetic elements as well as for high throughput screening is laid.

## AUTHOR CONTRIBUTIONS

**Gerrich Behrendt:** Conceptualization (equal); investigation (lead); methodology (equal); writing – original draft (equal). **Maria Vlachonikolou:** Investigation (supporting). **Helga Tietgens:** Investigation (supporting). **Katja Bettenbrock:** Conceptualization (equal); funding acquisition (equal); investigation (equal); methodology (equal); supervision (equal); writing – original draft (equal); writing – review and editing (equal).

## FUNDING INFORMATION

This work was funded by the German Federal Ministry of Education and Research (FKZ 031B0858) in the framework of the National Bioeconomy Strategy. We thank the Onassis Foundation Scholarship Programs for Hellenes and the Foundation for Education and European Culture for sponsoring contributing author Maria Vlachonikolou.

## CONFLICT OF INTEREST STATEMENT

None.

## Supporting information


File S1:

File S2:
