# Supplementary material for: Construction and comparison of different vehicles for heterologous gene expression in Zymomonas mobilis
Source: Microb Biotechnol. 2024 Jan 24;17(1):e14381. doi: 10.1111/1751-7915.14381 (PMC10832546; doi:10.1111/1751-7915.14381)
Supplement: Supplementary file 2 — File S2: [file MBT2-17-e14381-s001.docx]

**Supplementary File 2**

**Construction and comparison of different vehicles for heterologous gene expression in *Zymomonas mobilis***

Gerrich Behrendt^1^, Maria Vlachonikolou^1^, Helga Tietgens^1^, Katja Bettenbrock^1,*^

^1^ Analysis and Redesign of Biological Networks, Max Planck Institute for Dynamics of Complex Technical Systems, Sandtorstr. 1, 39106 Magdeburg, Germany

* Corresponding author: [bettenbrock@mpi-magdeburg.mpg.de](mailto:bettenbrock@mpi-magdeburg.mpg.de)

Content:

Figure S1: eGFP expression in cultures carrying different shuttle vectors with kanamycin resistance.

Figure S2: eGFP expression in cultures carrying different shuttle vectors with chloramphenicol resistance.

Figure S3: eGFP expression in cultures carrying different shuttle vectors with spectinomycin resistance.

| A) 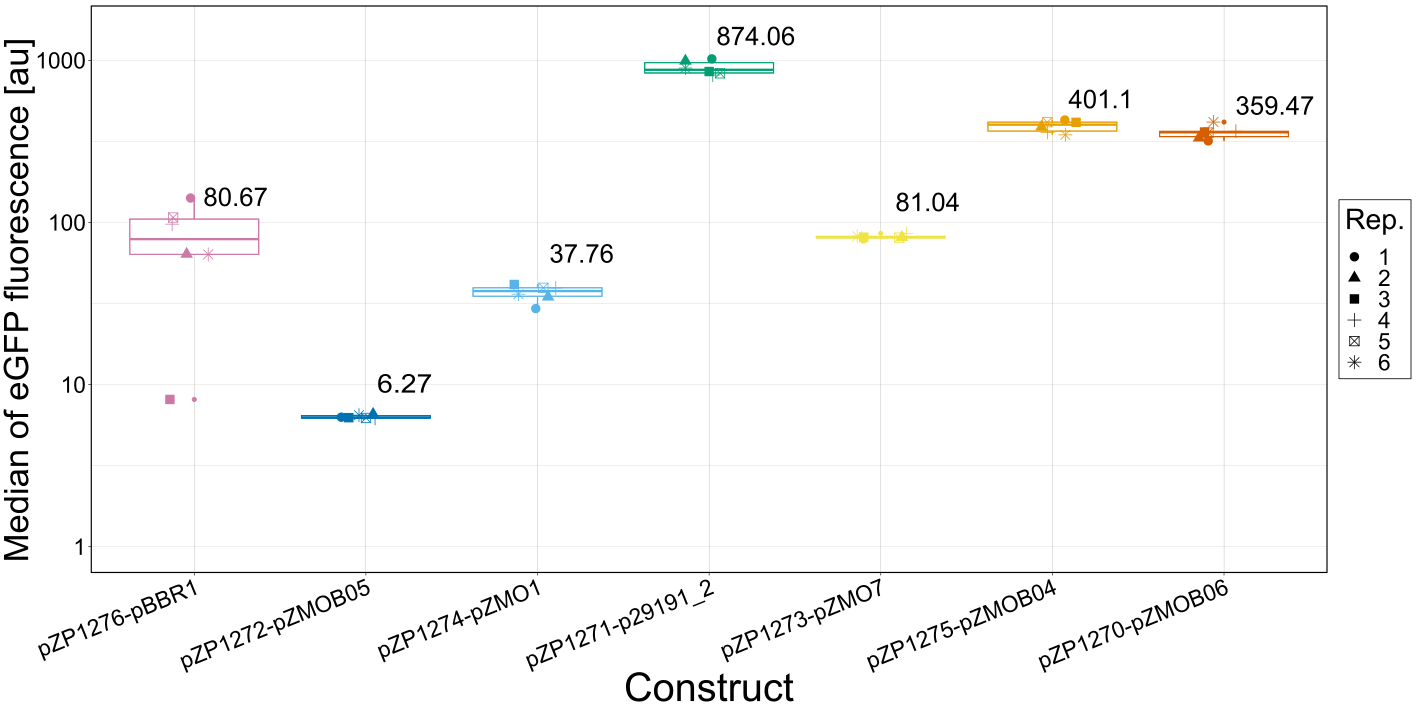 | B) 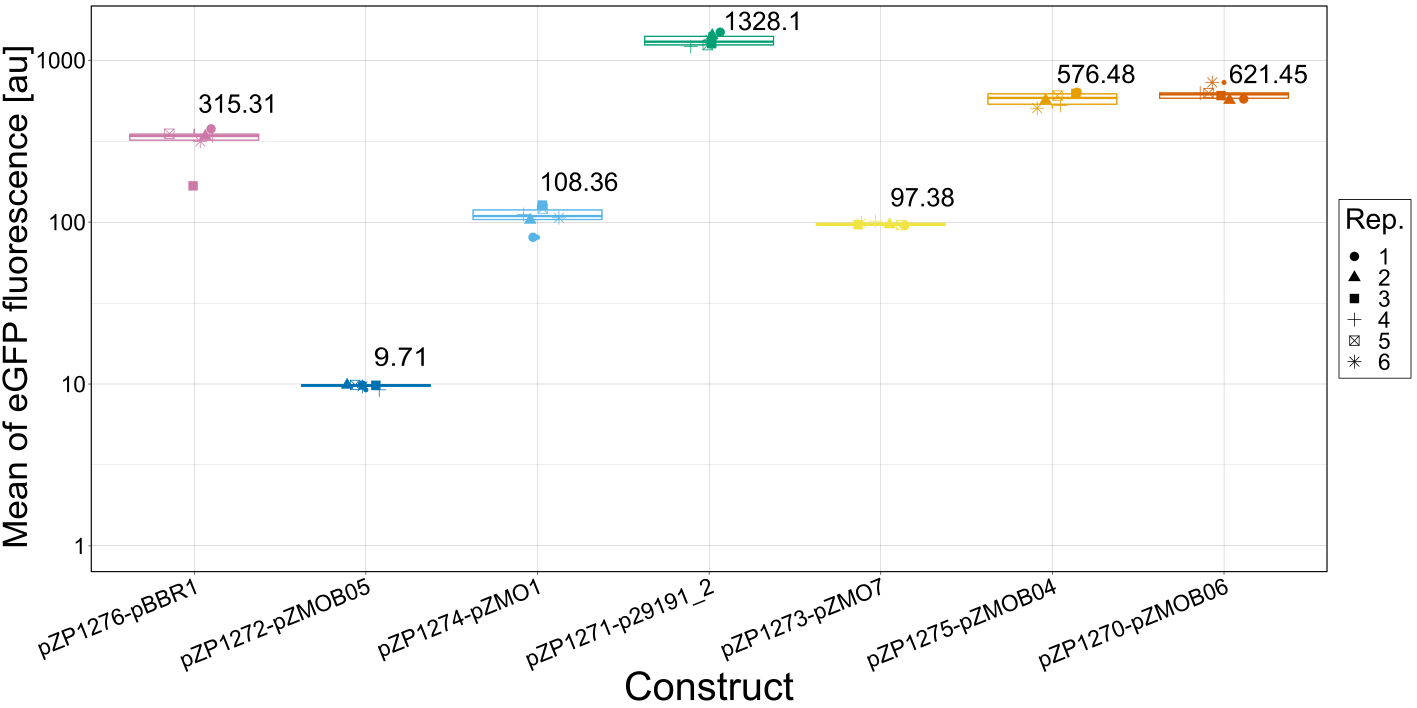 |
| --- | --- |
| C) 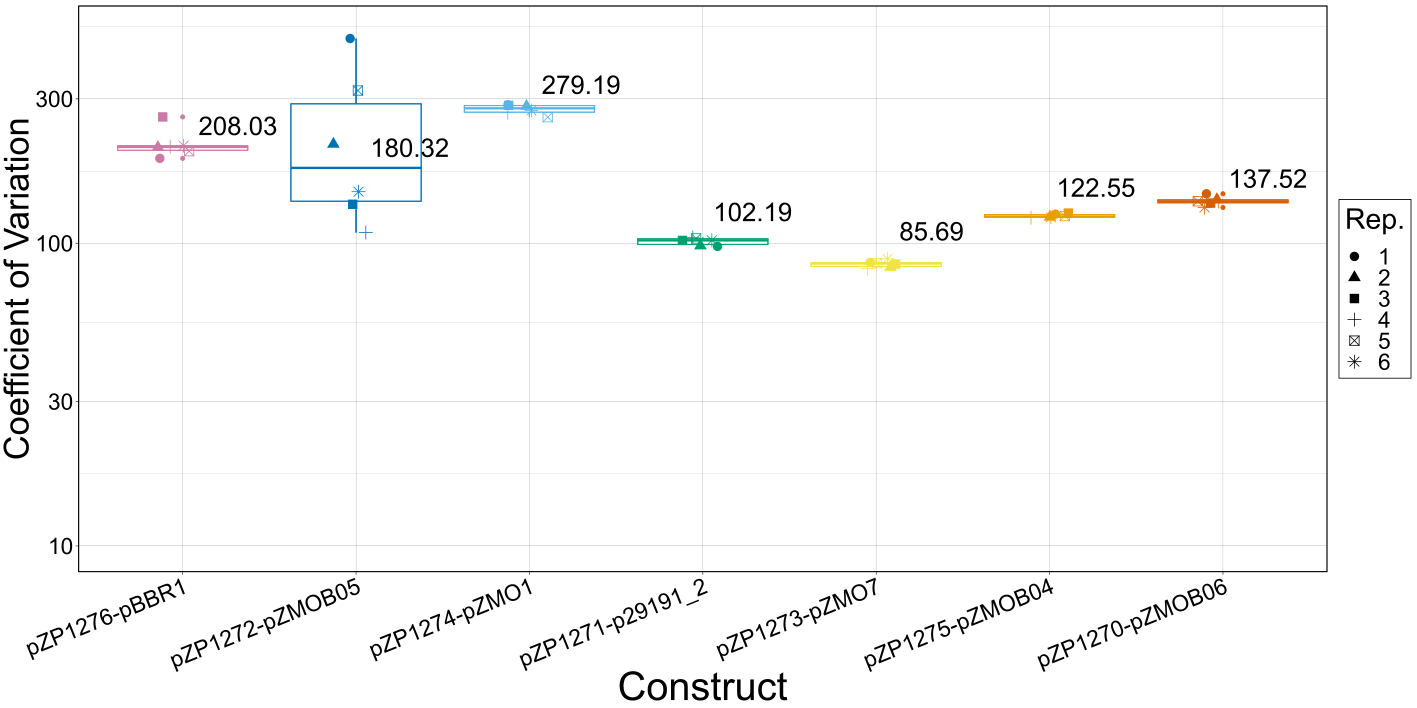 | D) 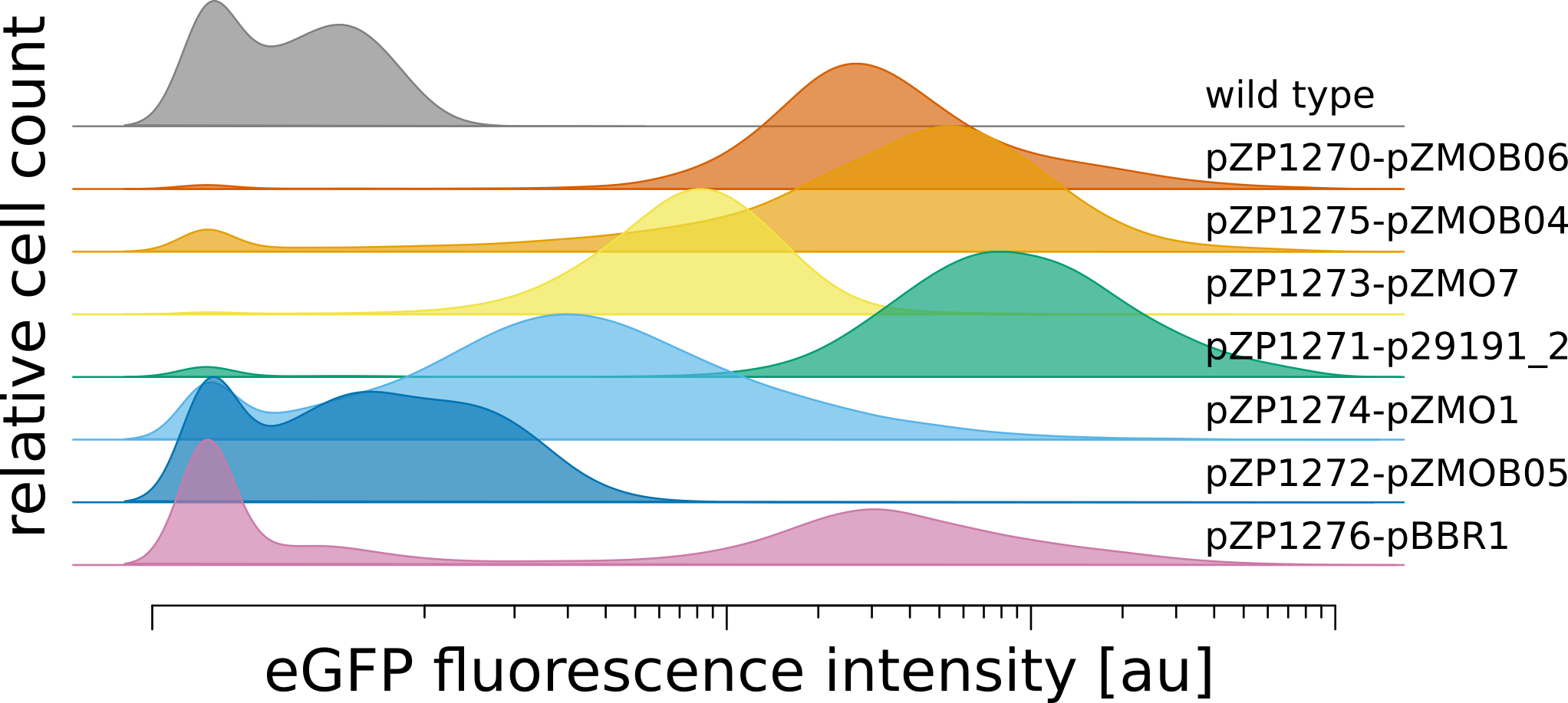 |

**Figure S1:** **eGFP expression in cultures carrying different shuttle vectors, with kanamycin resistance.** Box plots showing A) the medians, B) the means and C) the distribution (as CV) of eGFP fluorescence intensity using the same expression cassette in different shuttle vectors. Each point represents the eGFP fluorescence from a single cultivation of ATCC 31821 with the respective plasmids pZP1270 (pZMOB06 ori), pZP1271 (p29191_2 ori), pZP1272 (pZMOB05 ori), pZP1273 (pZMO7 ori), pZP1274 (pZMO1 ori), pZP1275 (pZMOB04 ori) and pZP1276 (pBBR1MCS2 ori). A total of six biological replicates per construct were measured. The median of all measurements per construct is displayed next to each box. D) Example histograms of the distribution of eGFP fluorescence intensity for 50.000 cells from ATCC 31821 with the respective expression plasmid.

| A)  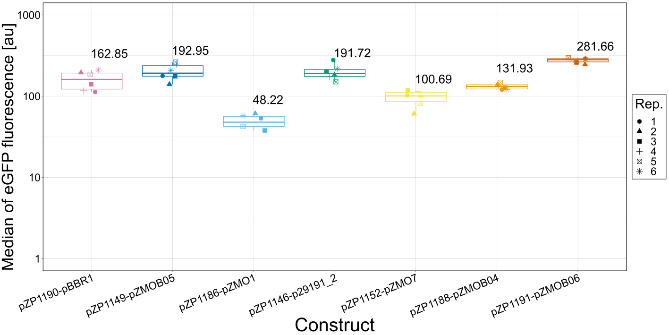 | B) 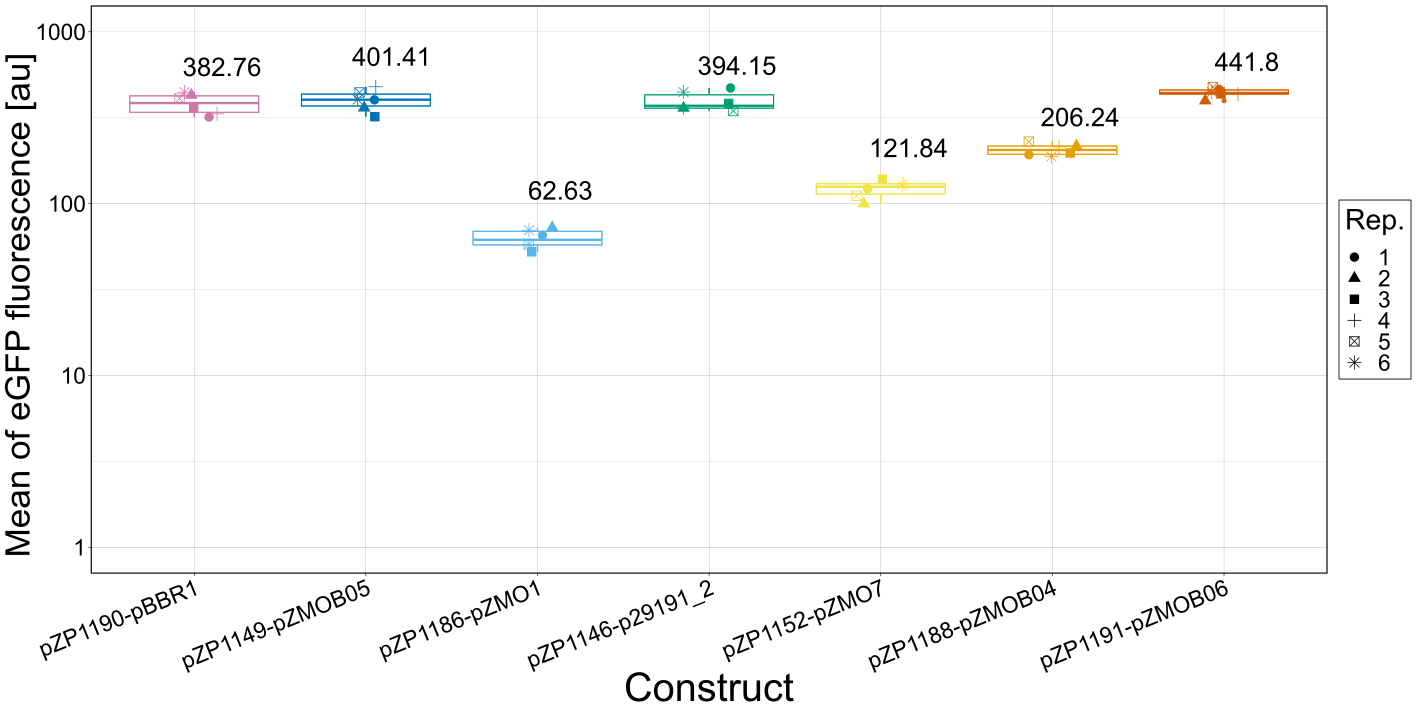 |
| --- | --- |
| C) 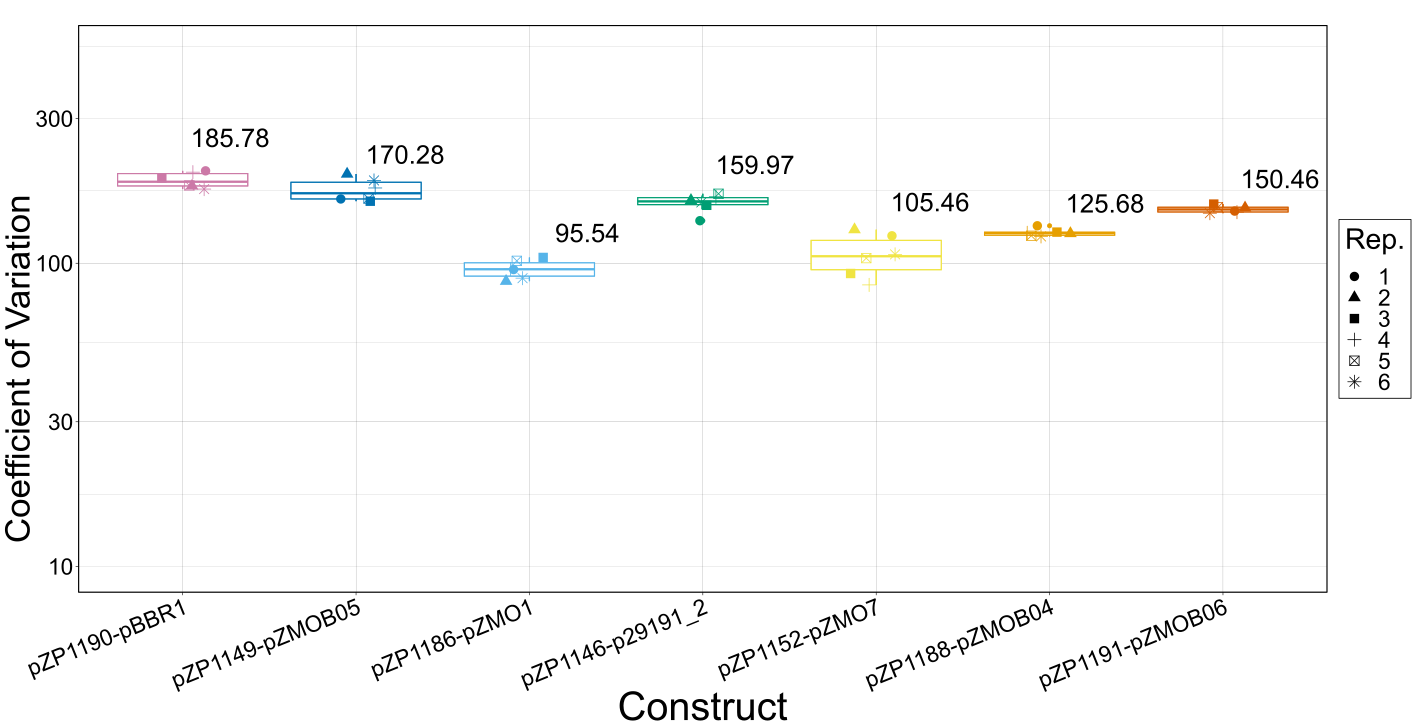 | D) 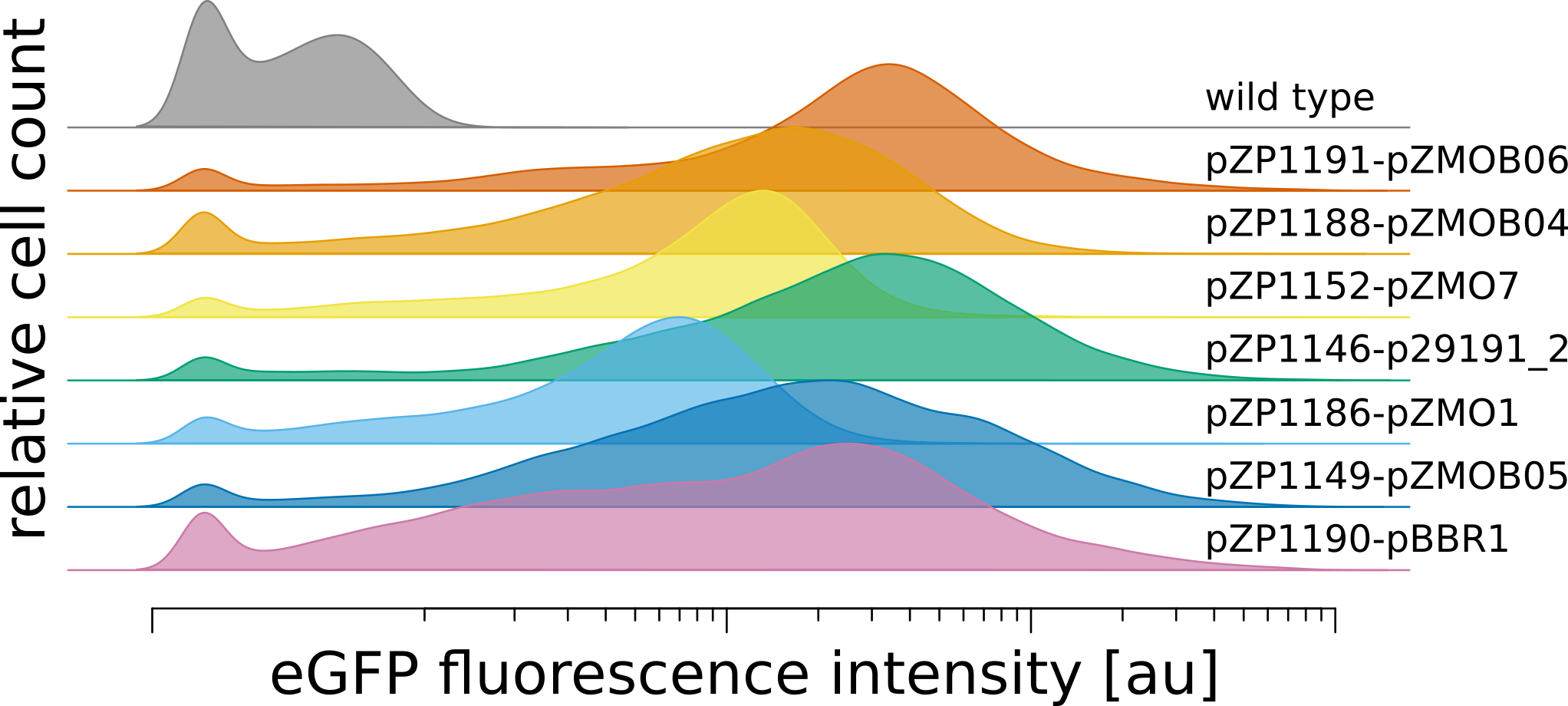 |

**Figure S2:** **eGFP expression in cultures carrying different shuttle vectors, with chloramphenicol resistance.** Box plots showing A) the medians, B) the means and C) the distribution (as CV) of eGFP fluorescence intensity using the same expression cassette in different shuttle vectors. Each point represents the eGFP fluorescence from a single cultivation of ATCC 31821 with the respective plasmids pZP1191 (pZMOB06 ori), pZP1146 (p29191_2 ori), pZP1149 (pZMOB05 ori), pZP1152 (pZMO7 ori), pZP1186 (pZMO1 ori), pZP1188 (pZMOB04 ori) and pZP1190 (pBBR1MCS2 ori). A total of six biological replicates per construct were measured. The median of all measurements per construct is displayed next to each box. D) Example histograms of the distribution of eGFP fluorescence intensity for 50.000 cells from ATCC 31821 with the respective expression plasmid.

| A) 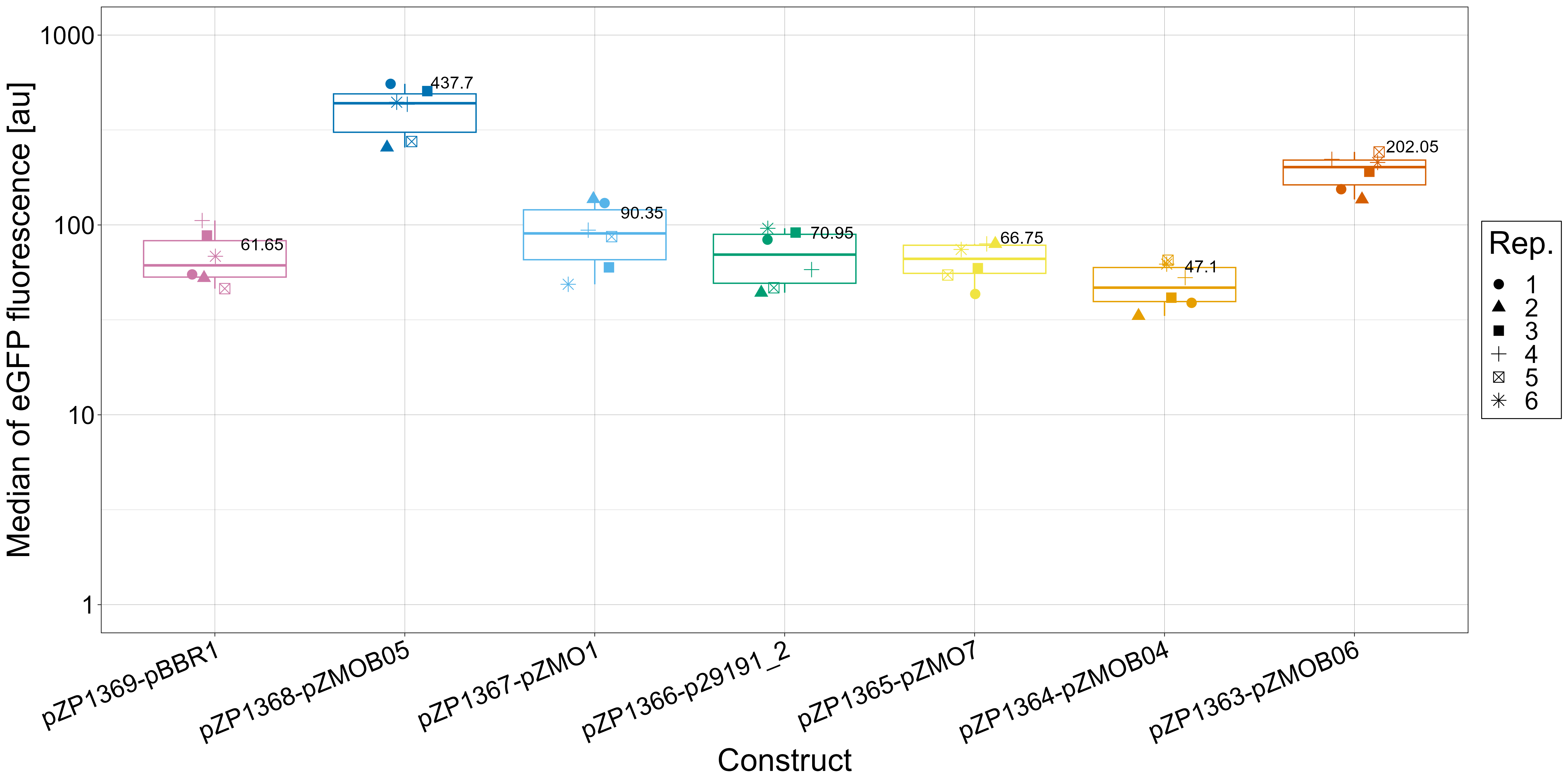 | B) 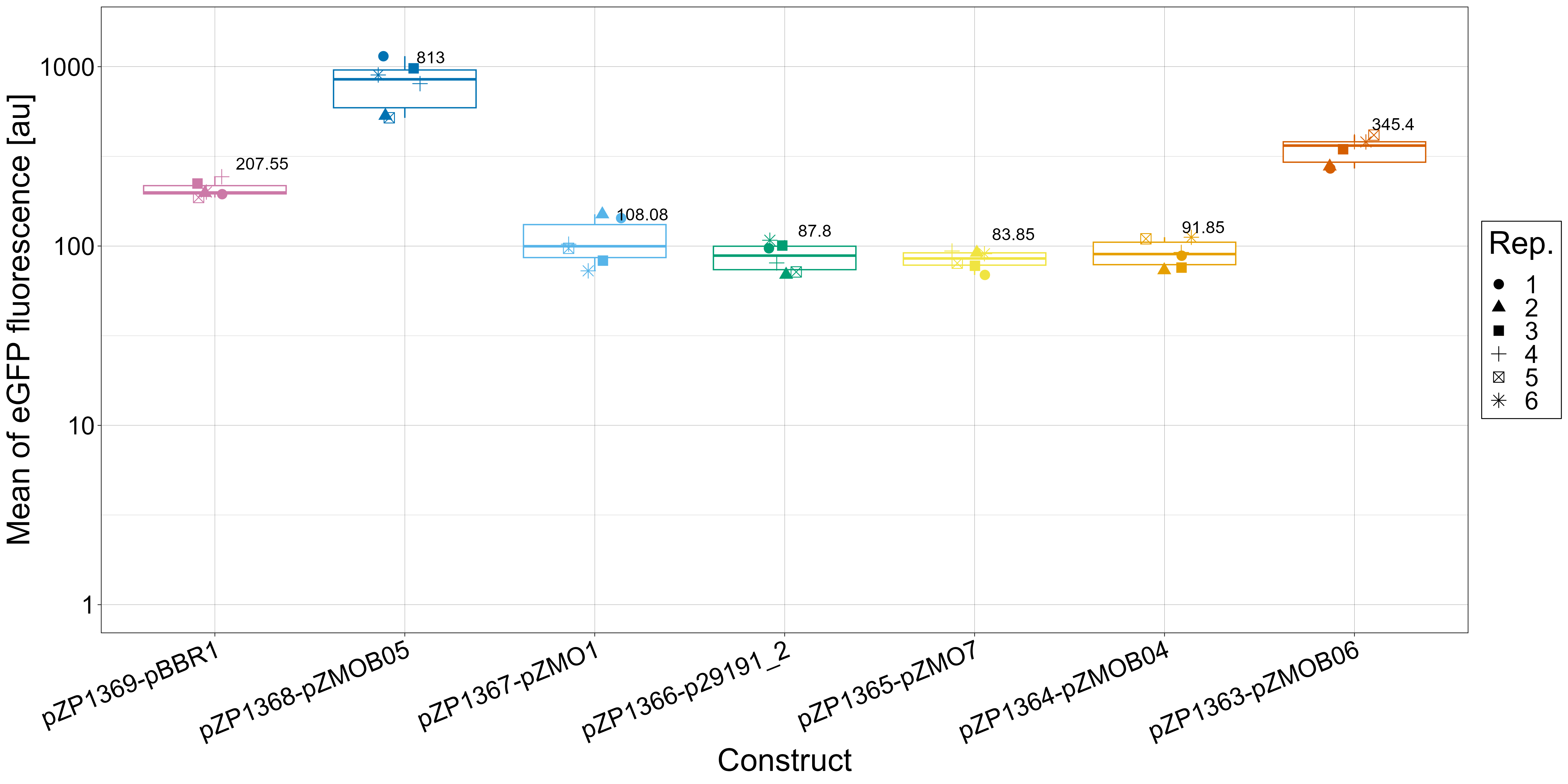 |
| --- | --- |
| C) 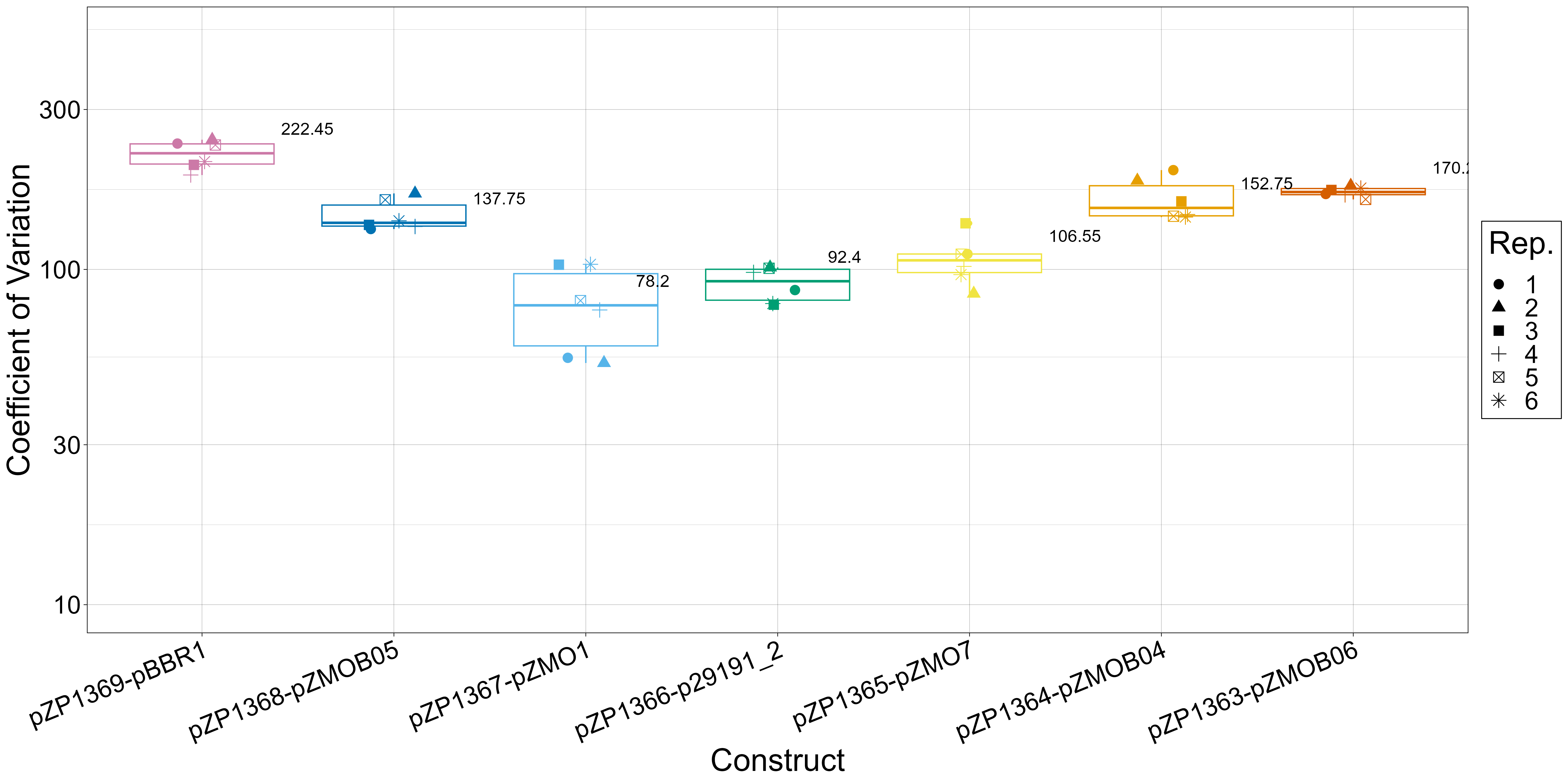 | D)  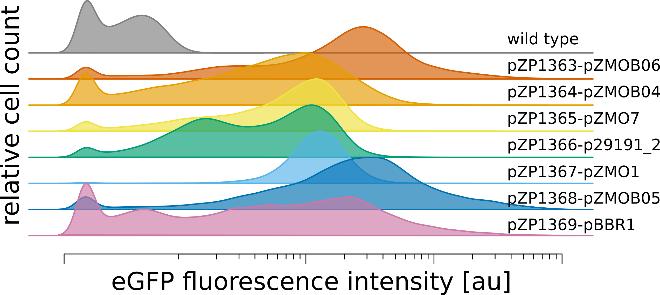 |

**Figure S3:** **eGFP expression in cultures carrying different shuttle vectors, with spectinomycin resistance.** Box plots showing A) the medians, B) the means and C) the distribution (as CV) of eGFP fluorescence intensity using the same expression cassette in different shuttle vectors. Each point represents the eGFP fluorescence from a single cultivation of ATCC 31821 with the respective plasmids pZP1363 (pZMOB06 ori), pZP1366 (p29191_2 ori), pZP1368 (pZMOB05 ori), pZP1365 (pZMO7 ori), pZP1367 (pZMO1 ori), pZP1364 (pZMOB04 ori) and pZP1369 (pBBR1MCS2 ori). A total of six biological replicates per construct were measured. The median of all measurements per construct is displayed next to each box. D) Example histograms of the distribution of eGFP fluorescence intensity for 50.000 cells from ATCC 31821 with the respective expression plasmid.
